# How Quorum Sensing Connects Sporulation to Necrotrophism in *Bacillus thuringiensis*

**DOI:** 10.1371/journal.ppat.1005779

**Published:** 2016-08-02

**Authors:** Stéphane Perchat, Antoine Talagas, Sandrine Poncet, Noureddine Lazar, Inès Li de la Sierra-Gallay, Michel Gohar, Didier Lereclus, Sylvie Nessler

**Affiliations:** 1 Micalis Institute, INRA, AgroParisTech, Université Paris-Saclay, Jouy-en-Josas, France; 2 Institute of Integrative Biology of the Cell (I2BC), CEA, CNRS, Univ. Paris-Sud, Université Paris-Saclay, Gif-sur-Yvette, France; The University of Texas-Houston Medical School, UNITED STATES

## Abstract

Bacteria use quorum sensing to coordinate adaptation properties, cell fate or commitment to sporulation. The infectious cycle of *Bacillus thuringiensis* in the insect host is a powerful model to investigate the role of quorum sensing in natural conditions. It is tuned by communication systems regulators belonging to the RNPP family and directly regulated by re-internalized signaling peptides. One such RNPP regulator, NprR, acts in the presence of its cognate signaling peptide NprX as a transcription factor, regulating a set of genes involved in the survival of these bacteria in the insect cadaver. Here, we demonstrate that, in the absence of NprX and independently of its transcriptional activator function, NprR negatively controls sporulation. NprR inhibits expression of Spo0A-regulated genes by preventing the KinA-dependent phosphorylation of the phosphotransferase Spo0F, thus delaying initiation of the sporulation process. This NprR function displays striking similarities with the Rap proteins, which also belong to the RNPP family, but are devoid of DNA-binding domain and indirectly control gene expression *via* protein-protein interactions in *Bacilli*. Conservation of the Rap residues directly interacting with Spo0F further suggests a common inhibition of the sporulation phosphorelay. The crystal structure of apo NprR confirms that NprR displays a highly flexible Rap-like structure. We propose a molecular regulatory mechanism in which key residues of the bifunctional regulator NprR are directly and alternatively involved in its two functions. NprX binding switches NprR from a dimeric inhibitor of sporulation to a tetrameric transcriptional activator involved in the necrotrophic lifestyle of *B*. *thuringiensis*. NprR thus tightly coordinates sporulation and necrotrophism, ensuring survival and dissemination of the bacteria during host infection.

## Author Summary

*Bacillus thuringiensis* is an entomopathogenic bacterium used worldwide as biopesticide. Its life cycle in insect larvae, which includes virulence, necrotrophism and sporulation, is regulated by cell-cell communication systems involving sensor proteins directly regulated by re-internalized peptide pheromones. After toxaemia caused by pore-forming Cry toxins, the PlcR sensor activates the production of virulence factors leading to insect death. *B*. *thuringiensis* then shifts to a necrotrophic lifestyle preceding sporulation. Previously, we showed that this process is regulated by the sensor NprR, which, in the presence of its cognate signaling peptide NprX, adopts a tetrameric conformation allowing its binding to specific DNA sequences and transcription of genes involved in survival of the bacteria in insect cadavers. Here, we demonstrate that, in the absence of NprX, NprR is a dimer, which negatively controls sporulation, independently of its transcription factor activity. We show that NprR prevents the phosphorylation of the phosphoprotein Spo0F and inhibits the phosphorylation cascade regulating sporulation. This demonstrates that NprX binding switches the bifunctional sensor NprR from a dimeric sporulation inhibitor to a tetrameric transcription factor. By establishing a close coordination between cell density, necrotrophism and sporulation, this communication system benefits a pathogenic bacterium feeding on death matter like *B*. *thuringiensis*. NprR is found in all strains of the *B*. *cereus* group, including *B*. *anthracis* and *B*. *cereus* involved in food poisoning. Our results may provide new insights for controlling the development and the survival of these undesirable bacteria.

## Introduction

Sporulating bacteria have developed a number of sophisticated mechanisms devoted to the temporal and spatial coordination of cell fate and gene expression. Regulatory mechanisms, as two-component systems, contribute to maintain this tight coordination by tuning gene expression and protein activation in response to a large variety of environmental stimuli including nutrient limitation and population density [1]. In Gram-positive bacteria, quorum sensing is a mode of cell-cell communication involving the secretion of diffusible signaling peptides recognized by the responder bacteria [2, 3]. These peptides elicit a response either directly, by binding to effector proteins or indirectly, by triggering a two-component phosphorelay.

The effectors of direct quorum-sensing systems are grouped in the RNPP protein family [4] named according to the first identified members: the Rap proteins of *Bacillus subtilis* [5, 6], the NprR and the PlcR transcriptional activators of *Bacillus cereus* and *B*. *thuringiensis* [7, 8] and the *Enterococcus faecalis* sex pheromone receptor PrgX [9, 10]. Recently the RNPP family was extended with the characterization of a new member identified in *Streptococci*, the SHP pheromone receptor Rgg [11]. These regulators are characterized by a conserved peptide-binding domain consisting of 6 to 9 copies of tetratricopeptide repeats (TPRs). Individual TPR motifs are degenerated sequences of ~34 residues composed of two anti-parallel α-helices. Their assembly results in a right-handed superhelix creating the peptide-binding pocket [12, 13]. Binding of the signaling peptides regulates the activity of their cognate quorum sensor [8, 10, 14, 15]. Except for the Rap proteins, RNPPs also contain an N-terminal helix-turn-helix (HTH)-type DNA-binding domain [16] and act as transcriptional regulators. The Rap proteins bind and regulate target proteins *via* an N-terminal alpha-helical domain replacing the HTH motif. In the complex with the target protein, this N-terminal domain displays a 3-helix bundle conformation, whereas it forms two additional TPR motifs in the peptide-bound form [17–20].

Interestingly, NprR contains both the HTH DNA-binding domain and the two Rap-like additional TPR motifs, and was thus proposed as an evolutionary intermediate in the RNPP family [4, 8]. We already demonstrated that in the presence of its cognate signaling peptide NprX, NprR acts as a major transcriptional regulator. The NprR-NprX complex regulates at least forty-one genes encoding proteins involved in the necrotrophic lifestyle of *Bacillus thuringiensis*, allowing the bacterium to survive in the insect cadaver [21]. A recent structure-function analysis demonstrated that the NprR-NprX complex adopts a tetrameric structure, suggesting that NprR binds simultaneously to two DNA-binding sites [22]. In the absence of bound peptide, NprR dissociates into dimers and loses its ability to bind DNA. Two types of interfaces respectively involved in dimer and tetramer formation were identified.

The sequence similarity with the Rap proteins led us to hypothesize that the dimeric form of apo NprR could act as a Rap-like indirect regulator of the sporulation process. The Rap family has been characterized in *B*. *subtilis* and *B*. *anthracis* and several members have been shown to prevent sporulation initiation by stimulating the dephosphorylation of the phosphotransferase Spo0F-P [5, 23, 24]. This phosphatase activity thus interrupts the phosphorelay regulating Spo0A, the key transcription factor, which controls early stationary phase and sporulation gene expression [25, 26].

It has been recently proposed that NprR is a positive activator of sporulation initiation and that the binding of the signaling peptide NprX activates the sporulation function of NprR [27]. These results, suggesting a different mechanism for Rap and NprR, were very surprising and in sharp contradiction with our data. As we show here, NprR acts as a Rap phosphatase, negatively affecting the commitment to sporulation in the absence of bound peptide and independently of its transcriptional activator function. NprR prevents expression of Spo0A-regulated genes and delays the initiation of the sporulation process by dephosphorylating Spo0F. In addition, we show that the dimeric apo form of NprR is highly flexible, as observed in the Rap proteins [18]. Finally, we propose a molecular mechanism for the NprR effect on sporulation. We explain how NprX binding promotes dissociation of Spo0F, formation of the NprR tetramer and DNA binding. Taken together, these results thus demonstrate that NprX regulates the switch from an indirect sporulation inhibitor function to a transcriptional activator function. By integrating an enzymatic activity and a regulatory function in a single molecule, NprR links adaptation properties and cell fate, thus allowing an accurate synchronization of important functions of the cell.

## Results

### NprR negatively affects sporulation and NprX binding inhibits this function

**Fig 1:**
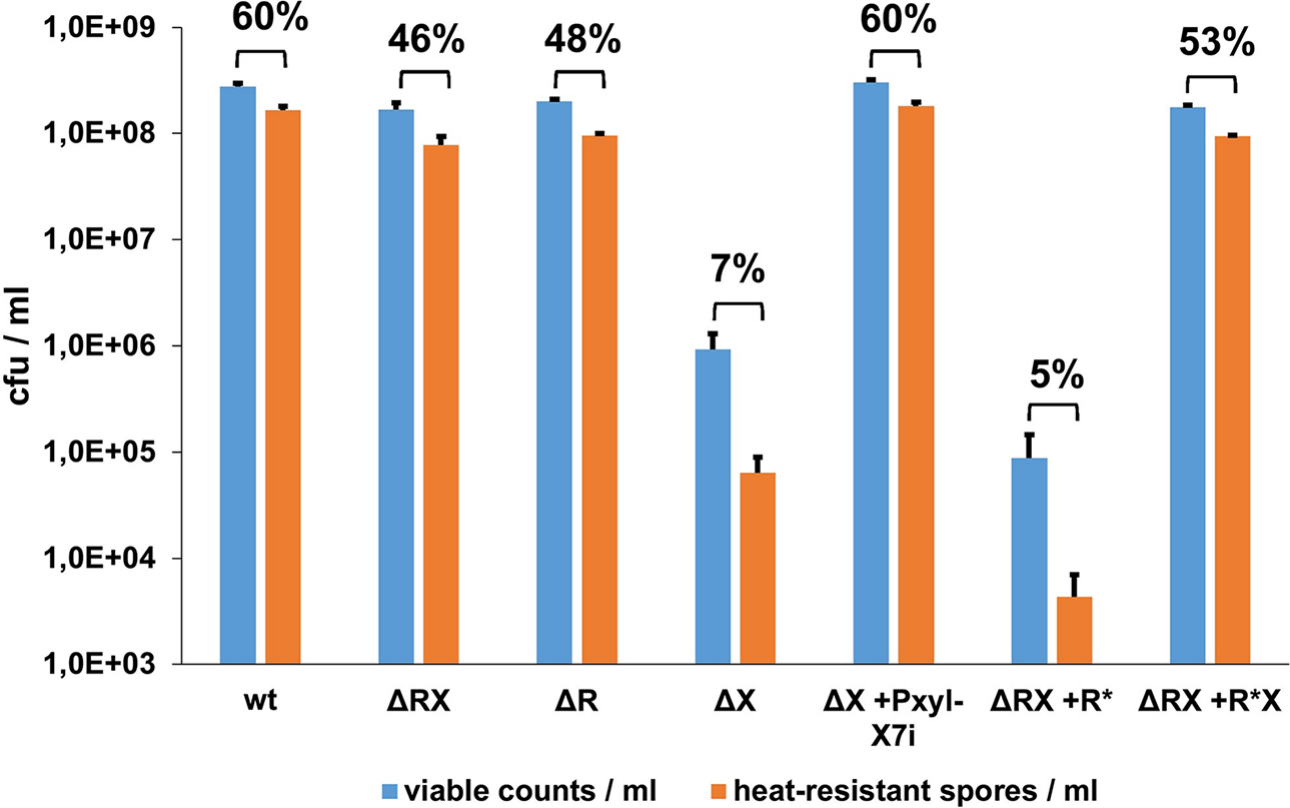
NprR negatively affects sporulation independently of its transcriptional activator function. Comparison between the number of viable bacteria (blue bars) and heat-resistant spores (orange bars) after 3 days of growth in LB medium at 30°C. The percentage of sporulation is indicated at the top of histogram bars. R* refers to the HTH-dead mutant of the protein. Error bars: Standard Error of the Mean (SEM). The experimental values are given in S1 Table.

In view of the similarity between the NprR and Rap proteins [4, 22], we investigated whether NprR could play a role in the sporulation process. We compared the sporulation rates of the parental *B*. *thuringiensis* 407 wild-type strain with the NprR/NprX-deficient strain (ΔRX), the NprR-deficient strain (ΔR) and the NprX-deficient strain (ΔX) (Fig 1). In the growth conditions used in this study (LB medium at 30°C), the OD_600_ at the onset of stationary phase (t0) was similar for all the strains (S1 Table). However, the percentage of sporulation of the ΔRX mutant strain was lower (46 ± 2.6%) than that of the wild type (60 ± 1.3%). A similar sporulation rate (48 ± 2.8%) was measured in the ΔR mutant strain demonstrating that NprX alone does not affect sporulation. In sharp contrast, the sporulation rate of the ΔX mutant strain was significantly reduced to 7 ± 1.5%, and the total number of viable bacteria of this mutant strain was 300-fold lower than in the wild-type strain. These results indicate that the significant proportion of bacteria, which did not enter into the sporulation process was unable to survive starvation. It results that the production of heat-resistant spores was reduced by 1200- and 2500-fold compared to the ΔRX and wild-type strains, respectively. These results suggest that NprR acts like a Rap-like protein negatively affecting sporulation in the absence of NprX peptide. The sporulation efficacy of a ΔX strain carrying the *X7i* gene (designated ΔX *amy*::X7i), which encodes the heptapeptide SKPDIVG corresponding to the minimal active form of NprX [8] was fully restored when the expression of *X7i* was induced (Fig 1). These results thus demonstrate that NprX inhibits the Rap-like function of NprR on sporulation and activates its transcriptional function.

To determine whether the role of NprR on sporulation is independent of its transcriptional activator function, we constructed an nprR* gene with a non-functional HTH domain. Based on the weight matrix established by Dodd and Egan for the evaluation of potential HTH motifs [28] we introduced the M20A, T21A and Q22A substitutions in the N-terminal HTH domain of the protein (NprR*). Moreover, sequence alignment with the HTH domain of PlcR and analysis of the PlcR/DNA interactions (PDB ID 3U3W) [29] showed that the MTQ motif is conserved and belongs to PlcR helix α2, which interacts with the sugar-phosphate backbone of the bound DNA, thus participating to stabilization of the complex (S1 Fig). The nprR* and nprR*-*nprX* genes were inserted at the *amy* locus of the ΔRX mutant and these strains were designated ΔRX *amy*::R* and ΔRX *amy*::R*X, respectively. No ß-galactosidase production was detected in the strain ΔRX *amy*::R*X harbouring P_*nprA’Z*_ (S2A Fig), thus demonstrating that the mutations introduced in the HTH domain of the NprR* protein result in the complete loss of its transcriptional activation function. We then tested the effect of the NprR* protein on sporulation efficiency (Fig 1). As described above for the ΔX strain, a sporulation-defective phenotype was observed with the ΔRX strain harbouring the nprR* gene (sporulation rate reduced from 46 ± 2.6% to 5 ± 0.6%). Moreover, the production of viable spores in the ΔRX *amy*::R* strain was 5-log lower than in the wild-type strain. As with native NprR, the sporulation efficiency of the strain producing NprR* was restored when NprX was produced. Taken together, these results demonstrate that the NprR protein with a non-functional HTH domain is unable to activate *nprA* expression but preserves its negative effect on sporulation in the absence of NprX. Therefore, the two NprR functions are dissociated: the effect of NprR on sporulation is not due to a pleiotropic effect of its functions as transcriptional activator.

### NprR prevents expression of Spo0A-regulated genes

**Fig 2:**
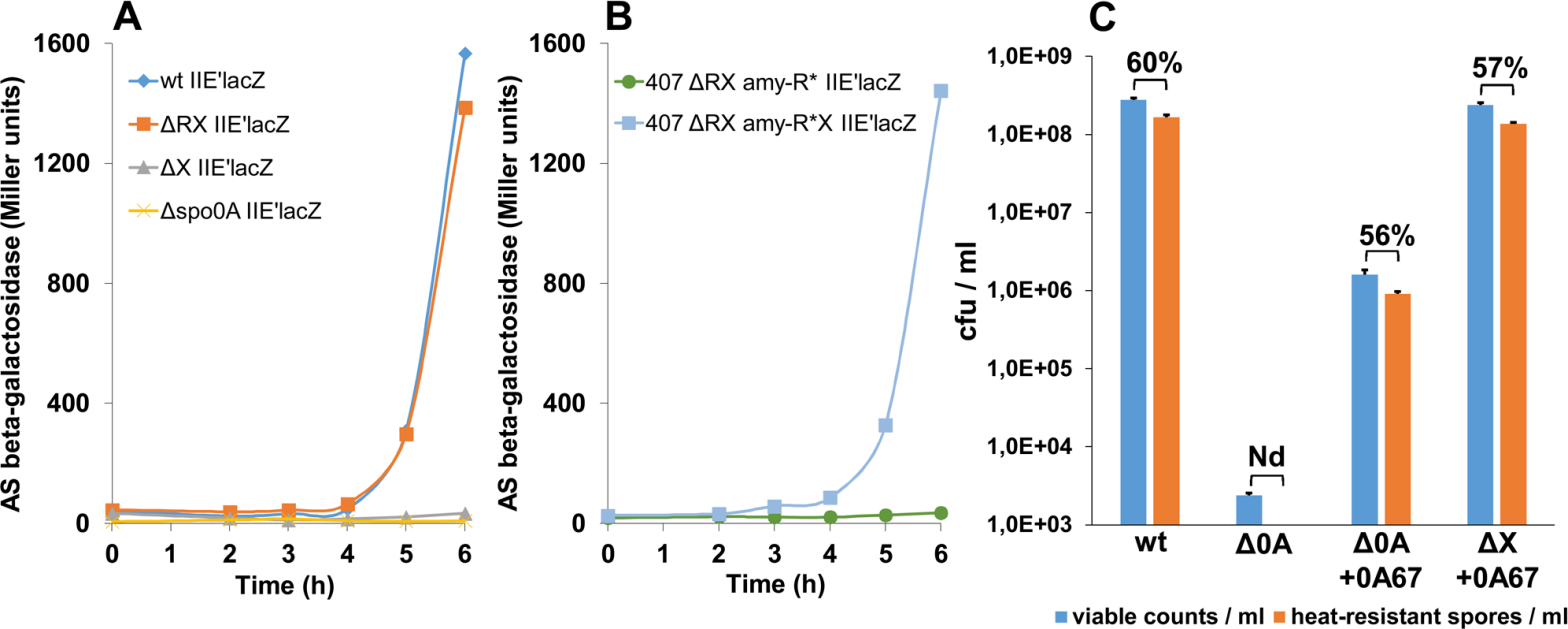
In the absence of NprX, NprR prevents expression of Spo0A-regulated genes. (A) Kinetics of *spoIIE* expression. β-Galactosidase activity of the strains *Bt* 407 (wt), *Bt* ΔRX, *Bt* ΔX and *Bt* Δspo0A harbouring the pHT304.18-*spoIIE*’*lacZ* plasmid. (B) NprR* lacking a functional HTH domain inhibits *spoIIE* transcription. β-Galactosidase activity of the strains *Bt* 407 (wt), *Bt* ΔRX *amy*::R* and *Bt* ΔRX *amy*::R*X harbouring the pHT304.18-*spoIIE*’*lacZ* plasmid. The bacteria were grown at 30°C in LB medium and time zero was defined as the onset of the stationary phase. (C) A constitutively active Spo0A initiates sporulation despite the absence of NprX. Comparison between the number of viable bacteria (blue bars) and heat-resistant spores (orange bars) after 3 days of growth in LB medium at 30°C. The percentage of sporulation is indicated at the top of histogram bars. Nd: not detected. Error bars: Standard Error of the Mean (SEM). The experimental values are given in S1 Table.

The initiation of sporulation is regulated by the phosphorylated state of Spo0A (Spo0A-P), the master regulator of sporulation [30]. To determine whether NprR, as the Rap phosphatases in *B*. *subtilis*, affects the phosphorylation of Spo0A in *B*. *thuringiensis*, we examined the activity of the *spoIIE* promoter, known to be under the direct control of Spo0A-P in *B*. *subtilis* [31]. A Spo0A box is present in the *spoIIE* promoter of *B*. *thuringiensis* and the *spoIIE’-lacZ* transcriptional fusion was not expressed in a 407 Δ*spo0A* mutant strain (Fig 2A), thus suggesting that *spoIIE* transcription is directly controlled by Spo0A in *B*. *thuringiensis* as in *B*. *subtilis*. We measured P_*spoIIE*_-directed ß-galactosidase expression in the wild type, ΔRX and ΔX strains. In the ΔX strain as in the Δ*spo0A* mutant strain, no *spoIIE* expression was observed whereas the ΔRX mutant behaved as the wild type (Fig 2A), suggesting that NprX inhibits the negative effect of NprR on *spoIIE* expression. We also verified that *spoIIE* expression was inhibited in the ΔRX strain complemented with nprR*, and that the *spoIIE* expression pattern was restored when NprX was produced (Fig 2B). Altogether, these results show that, in the absence of NprX, NprR inhibits sporulation independently of its transcriptional activator function by negatively affecting the transcription of Spo0A-regulated genes.

To investigate whether the role of NprR on sporulation depends on the phosphorylated state of Spo0A we used the Spo0A_Sad67_ mutant (designated 0A_67_), which is constitutively active independent of its phosphorylation state in *B*. *subtilis* [32]. Thus, the 0A_67_ bypasses the requirement of the phosphorelay to trigger sporulation. Due to the high level of conservation between the *B*. *thuringiensis* and *B*. *subtilis spo0A* genes [33], we assumed that such a mutation would have the same effect in these two *Bacillus* species. We verified the activity of the 0A_67_ protein by measuring the sporulation efficiency of the Δ*spo0A* strain harbouring the pHT-0A_67_. In the Spo0A-deficient strain, no heat-resistant spores were detected and the sporulation phenotype of the Δ*spo0A* harbouring the pHT-0A_67_ was partially restored when 0A_67_ was expressed (Fig 2C). The sporulation rate and the total production of heat-resistant spores were fully restored in the ΔX strain harbouring the pHT-0A_67_ plasmid (Fig 2C). These results show that the 0A_67_ protein, which bypasses the requirement of the phosphorelay, is able to trigger sporulation despite the absence of NprX. These results clearly demonstrate that the inhibition of sporulation by NprR relies on an action on the sporulation phosphorelay.

### The sporulation inhibitor activity of NprR relies on Spo0F dephosphorylation

**Fig 3:**
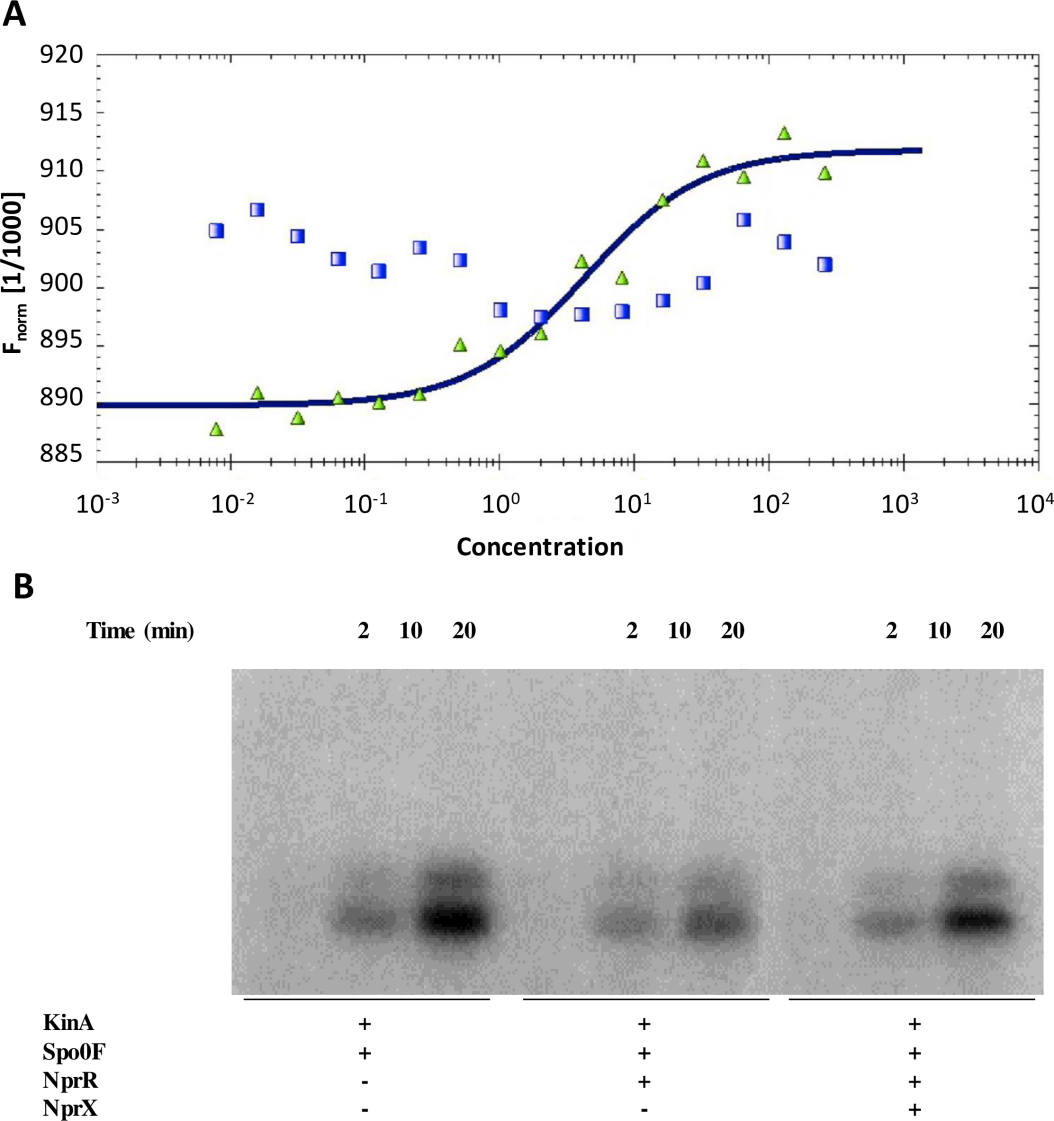
The sporulation inhibitor activity of NprR relies on Spo0F dephosphorylation. (A) Microscale thermophoresis analysis of the NprR-Spo0F interaction. The normalized NprR fluorescence F_norm_, i.e. the “hot” fluorescence divided by the “cold” fluorescence, is plotted on a linear y-axis in per mil (‰) against the total concentration of Spo0F on a log10 x-axis. The measurements were performed by using 20% LED power and 40% IR-laser power, in the presence (green triangles) and absence (blue squares) of NprX. (B) *In vitro* dephosphorylation of Spo0F-P by NprR. Purified *B*. *subtilis* Spo0F proteins (5.4 μM) was phosphorylated in a reaction containing *B*. *subtilis* KinA (0.1 μM) and [γ-32P]-ATP. Purified NprR protein and synthetic NprX-8 peptide (SSKPDIVG) were added at 10 μM and 57 μM final concentration, respectively. Time course experiments were carried out and aliquots withdrawn at the indicated time points.

In order to confirm that NprR acts like a Rap-like protein by targeting the phosphotransferase Spo0F, we used microscale thermophoresis to test the interaction of NprR with Spo0F from *B*. *thuringiensis* (Fig 3A). Our results confirmed direct binding between NprR and Spo0F with an apparent Kd value of about 5 μM. This interaction was lost in the presence of NprX, thus demonstrating the specificity of the NprR-Spo0F interaction and the regulatory role of the NprX peptide. The Rap proteins preferentially bind Spo0F-P and act as phosphatases [5]. Because no sporulation kinase has been characterized in the *B*. *thuringiensis* genome, we used the KinA-Spo0F system from *B*. *subtilis* to test if NprR displays the same mechanism, as already done to test the activity of Rap proteins from *B*. *anthracis* [24]. These results demonstrate that the KinA-dependent phosphorylation of Spo0F is reduced in the presence of NprR. Indeed, densitometry analysis revealed that, after 20 minutes of incubation in the presence of NprR, the phosphorylation of Spo0F was at least 3-fold lower compared to the control (band volume reduced from 64202 to 20287 arbitrary unit). Furthermore, addition of NprX restores the phosphorylation signal (Fig 3B). Taken together, these results thus demonstrate that NprR prevents the phosphotransfer from KinA to Spo0F and that peptide binding inhibits this activity.

### In the absence of NprX, apo NprR displays a highly flexible Rap-like structure

Our previous structural analysis demonstrated that the peptide-bound tetrameric crystal structure of NprR displayed a compressed conformation of the TPR-superhelix whereas the SAXS analysis of NprR suggested that the apo dimer would adopt a more extended conformation similar to the 3-helix bundle conformation of the Rap proteins [22]. To confirm this hypothesis and go further in the understanding of the sporulation inhibitor function of NprR, we needed to solve the crystal structure of the protein alone. As already observed in our previous crystallisation trials of the NprR-NprX complex [22], no crystals could be obtained using full-length NprR. We thus used the truncated NprR(ΔHTH) form of the protein, deleted of the HTH domains, which are known to be highly flexible in the absence of DNA. Because crystallisation trials are performed at very high protein concentration favouring the tetrameric form of NprR, we also used the (Y223A/F225A) mutant that has been shown to impair tetramer formation [22]. Crystals were finally obtained with the truncated double mutant protein NprR(ΔHTH)(Y223A/F225A). They diffracted up to 3.25Å resolution in space group P 21 21 21 with 2 molecules per asymmetric unit. The crystallographic phase problem was solved by the SAD method using a selenomethionine-labelled form of the protein. Atomic coordinates and structure factors have been deposited in the Protein Data Bank (PDB ID 5DBK).

**Fig 4:**
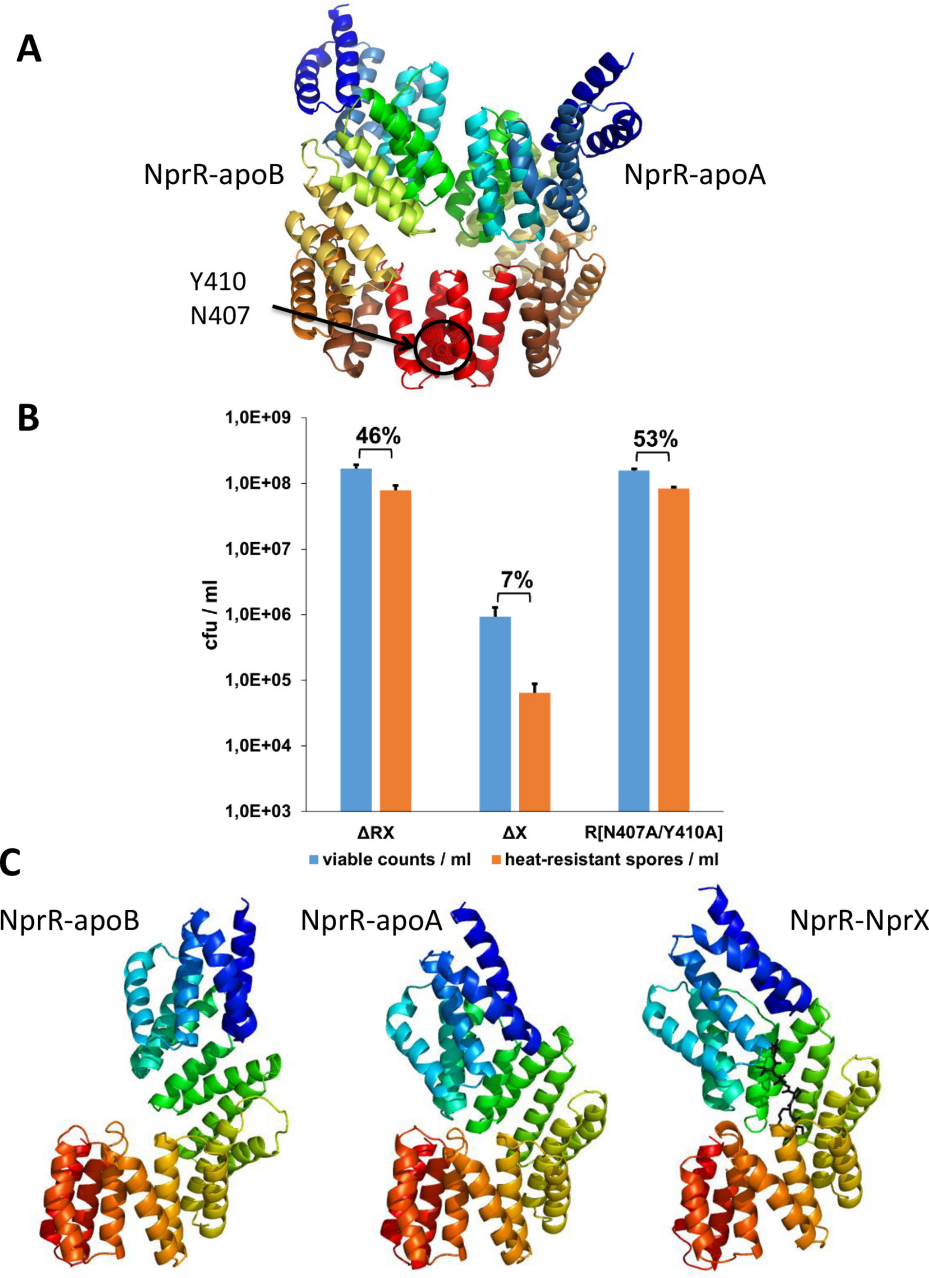
Apo NprR adopts a flexible Rap-like conformation. (A) The crystal structure of the NprR(ΔHTH)(Y223A/F225A) apo dimer is shown as cartoon with both subunits coloured as spectrum from blue (Nter) to red (Cter). The side chains of residues N407 and Y410 from the conserved C-terminal dimerization interface are highlighted by dots and labeled (B) Effect of NprR mutations N407A/Y410A on sporulation efficiency in the *Bt* ΔRX strain. The number of viable bacteria (blue bars) and heat-resistant spores (orange bars) was determined after 3 days of growth in LB medium at 30°C. The percentage of sporulation is indicated at the top of histogram bars. Error bars: Standard Error of the Mean (SEM). The experimental values are given in S1 Table. (C) Comparison of NprR-apoA and NprR-apoB with the closed conformation observed in the NprR-NprX complex (PDB ID 4GPK). The bound NprX peptide of 4GPK is highlighted as black sticks.

In both polypeptide chains contained in the asymmetric unit, the 18 α-helices and the connecting loops forming the 9 TPR motifs of NprR are well defined. Only some residues (up to seven) at the N- and C-terminal extremities of NprR(ΔHTH) as well as the C-terminal His-tag were not visible in the electron density. The two subunits (called apoA and apoB) display the right-handed superhelix conformation characteristic of TPR domains [13]. They are associated into a stable dimer (Fig 4A) characterized by an overall interface area of 1113Å^2^ and a solvation free energy gain Δ^i^G of -18 kcal/mol. The contacts involve 5 H-bonds and 2 salt bridges as well as hydrophobic interactions. In particular, the dimerization interface involving the C-terminal residues N407 and Y410, which was observed in the NprR/NprX complex, is conserved in the apo dimer. Interestingly, the NprR mutant N407A/Y410A, which has been shown to be monomeric and inactive as a transcription factor [22], has also lost its sporulation inhibitor activity (Fig 4B), demonstrating that NprR dimerization is essential for Spo0F binding.

Comparison of the two subunits of the apo NprR dimer demonstrated that, due to distinct crystal packing constraints, the pitch of the superhelix formed by the 9 TPR motifs of the protein is larger in apoB than in apoA (Fig 4C). This discrepancy, characterized by a Z-score of 7.9 and an rmsd distance of 1.99Å over 293 aligned Cα atoms, reflects the elasticity of the superhelix and suggests that, in solution and in the absence of NprX, the protein dynamically oscillates between distinct conformations. Comparison with the subunits of the symmetrical NprR-NprX tetramer (PDB ID 4GPK) (Fig 4C) showed that both apoA (Z-score = 7.7; rmsd = 2.31Å over 301 Cα atoms aligned with 4GPK-J) and apoB (Z-score = 5.1; rmsd = 2.74Å over 284 Cα atoms aligned with 4GPK-K) display a more extended conformation than the peptide-bound structure.

This flexibility of the TPR superhelix is also observed in the available structures of the Rap proteins. Peptide binding stabilizes a compressed form of the Rap superhelix similar to that observed in the NprR-NprX complex (PDB-ID 4GPK) [22] (Z-score = 9.3; rmsd = 2.26Å over 290 Cα atoms of chain 4GPK-F aligned with chain 4I9C-A of the RapF-PhrF complex [20]) (Z-score = 8,5; rmsd = 2.33Å over 288 Cα atoms of chain 4GPK-K aligned with chain 4GYO-A of the RapJ-PhrC complex [19]). On the other hand, binding of the Rap protein target stabilizes the 3-helix bundle conformation (structures of the RapH-Spo0F, PDB ID 3Q15 [18] and RapF-ComA, PDB ID 3ULQ [17] complexes). Interestingly, the available structures of the apo form of Rap proteins display distinct conformations. Apo RapI (PDB ID 4I1A, [19]) displays an extended TPR superhelix conformation similar to NprR apo-B (Z-score = 6.4; rmsd = 3.30Å over 307 aligned Cα atoms aligned with chain 4I1A-B). In turn, apo RapF displays the 3-helix-bundle conformation (PDB ID 4I9E, [20]) (S3 Fig). This demonstrates that the conformational change is not induced by protein binding but intrinsic to the flexibility of the TPR-superhelix.

The structural similarity between NprR and the Rap proteins, and in particular the conserved flexibility of the TPR superhelix, strongly suggests that NprR could adopt a 3-helix bundle-like conformation to carry its Rap-like phosphatase activity on Spo0F-P. NprR would thus follow the same molecular mechanism than the Rap phosphatases, despite the additional presence of the HTH domain at the N-terminus. We thus propose that NprR apoA and apoB represent two intermediate conformations of the protein, between the compressed TPR superhelix of the peptide-bound complex and the 3-helix bundle conformation of the Spo0F-bound form.

### Conserved key residues are involved in both functions of NprR

**Fig 5:**
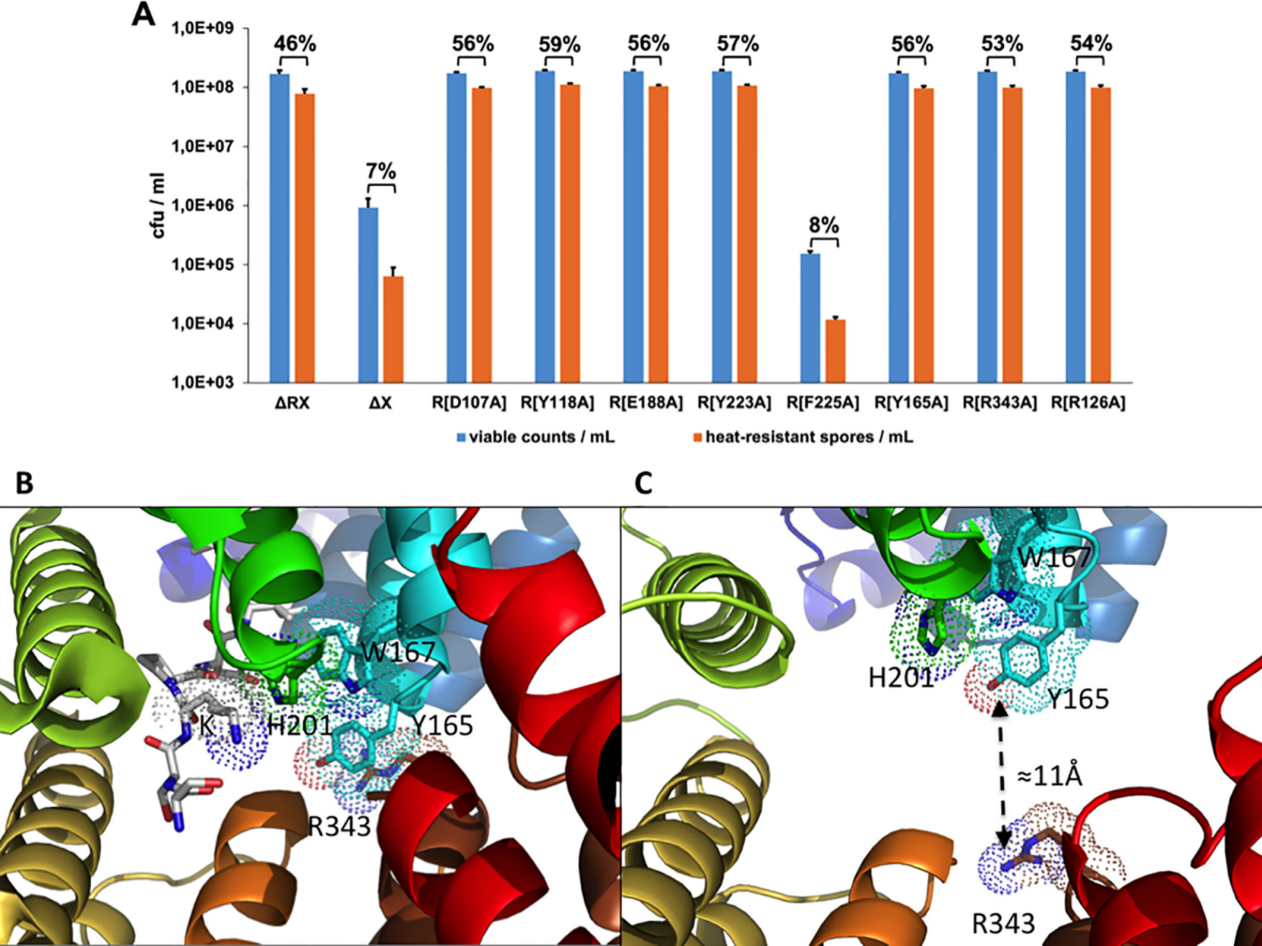
The same key residues are involved in the two functions of NprR. (A) Effect of NprR mutations in sporulation efficiency in the *Bt* ΔRX strain. The number of viable bacteria (blue bars) and heat-resistant spores (orange bars) was counted after 3 days of growth in LB medium at 30°C. The percentage of sporulation was indicated at the top of histogram bars. Error bars: Standard Error of the Mean (SEM). The experimental values are given in S1 Table. (B) Close view of the peptide-binding site in the NprR-NprX complex (PDB ID 4GPK). The bound peptide is shown as sticks coloured by atom type. Its K residue is labelled and highlighted by dots as well as NprR residues H201, W167, Y165 and R343. (C) Close view of the peptide-binding site in the NprR-apoB conformation. The increased distance between the NprR residues Y165 and R343 is indicated.

Structure based-sequence alignments (S4 Fig) showed that the RapH residues Q47, F58, E137 and Y175, which have been shown to be directly involved in Spo0F binding [18], correspond to NprR residues D107, Y118, E188 and Y223, respectively. We mutated these NprR residues into alanine and tested the ability of the resulting mutant proteins to inhibit sporulation. The four point mutations abolished the negative effect of NprR on sporulation (Fig 5A), confirming a Rap-like Spo0F binding mode. Because Y223, together with residue F225, is part of the interface stabilizing the tetrameric conformation of the NprR-NprX complex [22], we also mutated F225, which is not conserved in RapH, and measured its activities. Both single mutants NprR(Y223A) and NprR(F225A) had lost their transcriptional activity (S2B Fig) but the NprR(F225A) mutant retained its sporulation inhibitor activity (Fig 5A). Taken together these results demonstrate that the NprR residue Y223 is alternatively involved in tetramer formation and in Spo0F interaction, depending on the presence or absence of bound peptide, respectively, whereas F225 is only involved in stabilization of the DNA-binding tetramer. We thus propose that: i) Spo0F binding most probably stabilizes apo NprR in a Rap-like 3-helix bundle conformation, and ii) NprX binding stabilizes the compressed conformation of the TPR superhelix compatible with tetramer formation and DNA binding [22]. However, since only the truncated form of the protein could be crystallized, the position of the additional N-terminal HTH-domain of NprR remains unknown.

In our previous structural study of the NprR-NprX complex we showed that peptide binding involves residues from TPR-2 (residue R126) to TPR-7 (residues N306, D309). In particular, the importance of a stacking interaction between the K residue of the bound SSKPDIVG octapeptide and the NprR residues H201 and W167 has been demonstrated [22]. We show here that this interaction further induces an additional stacking interaction between residues Y165 from TPR-3 and R343 from TPR-8 (Fig 5B). Dissociation of the peptide releases this constraint, resulting in a dynamic flexibility of the TPR superhelix. In the relaxed conformation of the superhelix observed in NprR apoB, residues Y165 and R343 are about 11Å apart (Fig 5C). The role of the Y165-R343 interaction has been investigated by mutational analysis. A ß-galactosidase assay showed that these residues are necessary for the transcriptional activity of NprR (S2C Fig), thus confirming their role in stabilization of the closed transcription factor conformation of the protein. Interestingly, the sporulation inhibitor activity of the NprR mutant proteins (Y165A) and (R343A) was also abolished (Fig 5A), suggesting that these residues could also play a role in regulating Spo0F binding.

Furthermore, mutating into alanine residue R126, which had been shown to be essential for peptide binding [22], abolishes the inhibitory effect of NprR on sporulation (Fig 5A), suggesting that this residue is also alternatively involved in Spo0F binding.

## Discussion

Unlike the results published by Cabrera and colleagues [27] claiming that NprR has a positive effect on sporulation, our results demonstrate that NprR is a bifunctional regulator repressing sporulation in the absence of its cognate peptide NprX and acting as a transcriptional activator in the presence of peptide. These findings are in agreement with previous studies suggesting that NprR is an evolutionary intermediate between the Rap and the transcriptional activators of the RNPP family [4, 8].

We show that the mechanism involved in the negative control of sporulation is due to a second regulatory function of NprR, independent of its transcriptional activator function. Moreover, expression of the Spo0A-regulated genes *spoIIE* and *spoIIG* is prevented in the NprX-deficient strain, demonstrating that NprR affects Spo0A phosphorylation in the absence of signaling peptide. This new function of NprR is similar to the function of the Rap phosphatases, which interact with Spo0F-P in the absence of the Phr peptides [19, 34]. Dephosphorylation of Spo0F-P disrupts the phosphorelay therefore blocking the activation of Spo0A and the commitment to sporulation. Here we demonstrate that NprR binds to Spo0F and prevents its KinA-dependent phosphorylation, thus repressing the expression of Spo0A-regulated genes and finally inhibiting sporulation. This activity is relieved by NprX binding. According to these results, the NprR-NprX system thus functions as the Rap-Phr systems. However, the sporulation efficiency of the NprR/NprX-deficient strain was slightly lower than the wild type strain contrary to the Rap/Phr-deficient strains in *B*. *subtilis* and *B*. *anthracis* [24, 35]. The transcriptional activity of the NprR/NprX complex regulating genes coding for several degradative enzymes providing the nutrients required for bacterial survival and sporulation [21] could explain this result.

In the Rap proteins, the 3-helix bundle conformation has been shown to be required for binding of the target protein, Spo0F or ComA [19]. Our structural analysis suggests that NprR, despite the presence of the additional HTH domain at the N-terminus, most probably undergoes a similar conformational change for Spo0F binding.

It is also noteworthy that, in the Rap proteins where dimerization is not universally observed in solution [17, 23], the analysis of the assemblies and interfaces using PISA [36] revealed that all available crystal structures display a dimerization mode similar to NprR (S3B Fig). This interface could be less important and weaker in the Rap proteins than in the other RNPP regulators, which require dimerization for DNA binding. However, detailed analysis of the dimerization interface of RapF revealed that it involves residues equivalent to NprR residues, which have been shown to play important roles in dimerization. In particular, the NprR residues H205 and H206 are equivalent to RapF residues Y158 and F159. In the 3-helix bundle conformation of the RapF dimer (PDB ID 4I9E), symmetrical F159 residues are in stacking interactions and Y158 forms a sandwich interaction involving Y117 and R115 from the neighbouring subunit (S5A Fig). In the peptide-bound TPR conformation of RapF (PDB ID 4I9C), the interaction network between symmetrical Y158 and F159 residues is modified (S5B Fig) and Y117 now interacts with the bound peptide and Y153 (S5C Fig). This situation is reminiscent of what is observed in the NprR-NprX complex with residues W167 and H201 (Fig 5A). In addition, R115 interacts with D297, thus stabilizing the TPR conformation of RapF (S5C Fig), as observed in the NprR-NprX complex between residues Y165 and R343 (Fig 5C). Moreover, the conserved tryptophan residue corresponding to RapH W17 that has been shown to be important for the folding of the 3-helix bundle conformation [18] is conserved and corresponds to NprR W79 (S4 Fig). This structural comparison further supports the hypothesis that NprR and the Rap proteins would share the same mechanism and that NprR could adopt the 3-helix bundle conformation for Spo0F binding.

**Fig 6:**
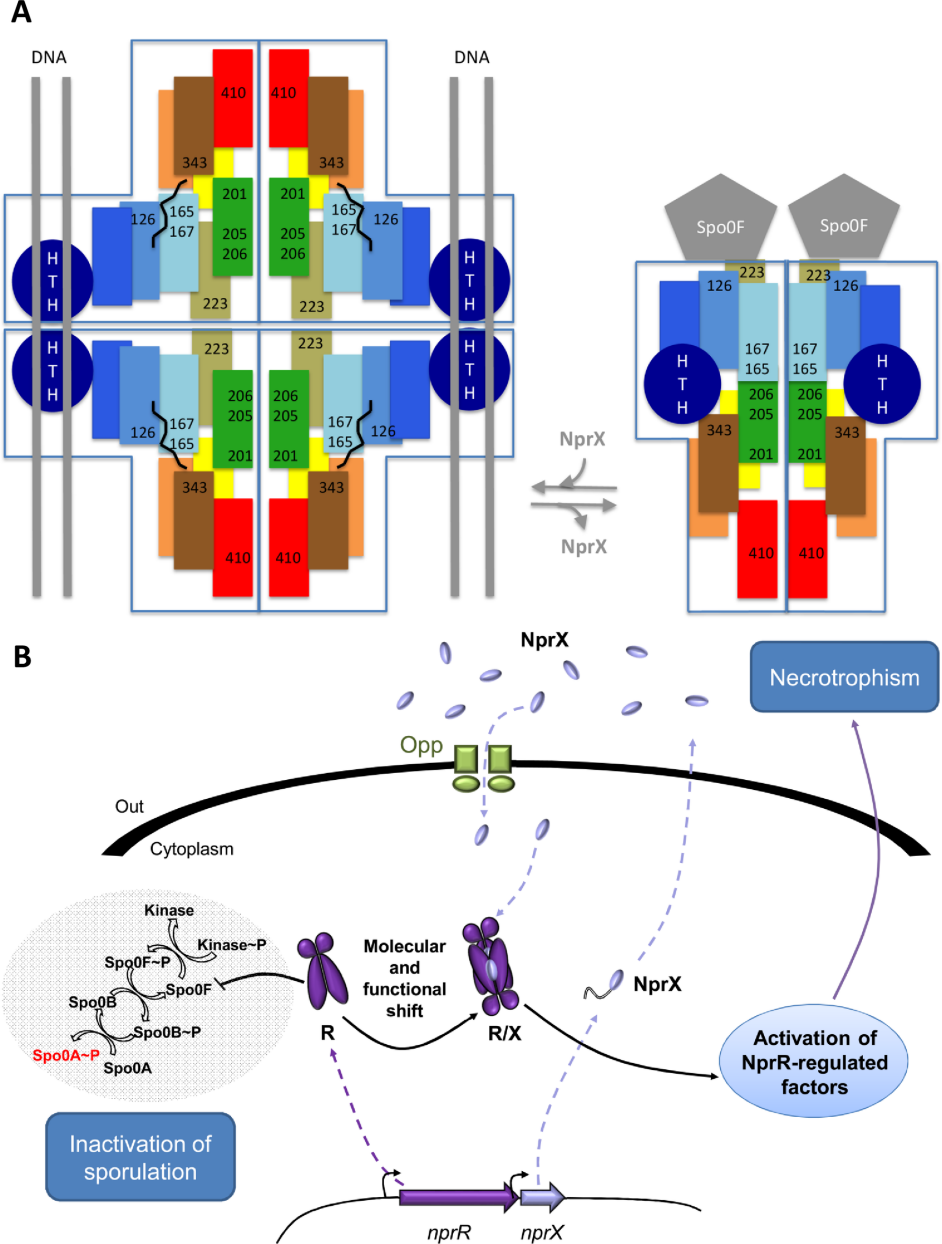
Proposed molecular mechanism of the bifunctional NprR/NprX quorum sensing system. (A) Structural switch. In the presence of the signaling peptide NprX, the TPR superhelix is locked in a closed conformation favouring tetramerization and formation of two DNA binding sites. In the absence of NprX, the TPR superhelix is released, inducing dissociation of the tetramer into flexible dimers capable of a drastic conformational change resulting in the 3-helix bundle conformation compatible with Spo0F binding. (B) Functional switch. The binding of NprX induces a switch from one activity (NprR = non-transcriptional inhibitor) to another activity (NprR-NprX = transcriptional activator). The regulatory protein NprR and its signaling peptide NprX are produced in the cytoplasm. First, NprR is present in the dimeric apo form, which binds to Spo0F and inactivates the initiation of the sporulation process by conserving Spo0A in an inactive state. NprX is secreted, extracellularly processed presumably as an octapeptide (SSKPDIVG) and reimported within the bacterial cell. When the intracellular concentration of NprX increases peptide binds to NprR, which shifts into the closed conformation incompatible with Spo0F binding allowing the phosphorylation of Spo0A and thus activating the commitment to sporulation. Concomitantly, the NprR-NprX complex tetramerizes, binds to DNA and activates the transcription of the NprR-regulated genes involved in necrotrophism.

However, understanding how this bifunctional quorum sensor accommodates the presence of additional HTH domain at the N-terminus will require further investigations. We propose that NprR residue R343, which has been shown to be essential for the sporulation inhibitor function of the protein, could be involved in stabilization of the HTH domain in the complex with Spo0F. The drastic conformational change resulting in the 3-helix bundle conformation indeed brings the equivalent residue RapF-D297 in the vicinity of helix α1 N-terminus, suggesting that NprR-R343 could interact with the N-terminal HTH-domain of NprR missing in the crystal structure. In the meantime, we propose a molecular regulatory mechanism for NprR (Fig 6A) in which important residues are directly and alternatively involved in the transcription factor and sporulation inhibitor functions of NprR. In particular, Y223 is alternatively involved in tetramerization and Spo0F binding. Similarly, we propose that residues Y165 and W167 alternatively stabilize the NprX-induced tetrameric conformation and the 3-helix bundle dimer compatible with Spo0F binding.

From a physiological point of view, we propose (Fig 6B) that during the transition phase between the end of exponential growth and the onset of sporulation, NprR is predominantly non-associated with NprX and present in a flexible dimeric form compatible with the conformational change required for Spo0F binding. The sporulation kinase is thus unable to phosphorylate the NprR-bound Spo0F. The phosphorylation cascade is inhibited and Spo0A is kept in an inactive state. As a consequence, the PlcR regulon is maintained, resulting in the production of extracellular virulence factors such as haemolysins, degradative enzymes and enterotoxins [37]. This allows the bacteria to be maintained in a virulence state ending by host death [3]. At this stage, the bacterial density in the host cadaver increases to a plateau and the intracellular concentration of matured-NprX is sufficient to lock NprR in the tight superhelix conformation incompatible with Spo0F binding, thus activating the sporulation phosphorelay. Concomitantly, the NprR-NprX complex tetramerizes and binds to DNA, thus activating the transcription of the NprR-regulated genes involved in the necrotrophic lifestyle of the bacteria [21].

Recently, a study on the dynamics of cell differentiation in *B*. *thuringiensis* population during growth in various conditions demonstrated that commitment to sporulation depends on the activity of NprR as a transcriptional activator [38]. This is in agreement with the new role of NprR-NprX in the modulation of the Spo0A phosphorylation rate suggesting that the Spo0A-P concentration required to initiate the sporulation process is reached only in bacteria engaged in the necrotrophism. This mechanism coupling necrotrophism with spore formation would ensure the survival and the dissemination of the bacteria during the infection process.

Our work thus revealed unexpected properties of NprR and NprX concerning their role in the developmental program of bacteria belonging to the *B*. *cereus* group. Altogether our results indicate that the NprR-NprX regulation system functions as a typical Gram-positive quorum-sensing system in which NprX is the signaling peptide. However, a striking difference between NprR-NprX and other known systems, including PlcR-PapR and Rap-Phr, is the dual function of the regulator protein: 1) In the absence of NprX, NprR prevents Spo0A to be phosphorylated by inhibiting phosphorylation of Spo0F; and 2) In the presence of bound NprX, NprR acts as a transcriptional regulator. Therefore, NprX acts as a switch, which toggles NprR from one activity (NprR = developmental function) to another (NprR-NprX = transcriptional activator). These two regulatory activities control completely distinct pathways: NprR combined to NprX activates the transcription of a set of genes involved in the necrotrophic lifestyle whereas NprR alone inhibits sporulation events. Bifunctional regulators, also designated as moonlighting proteins, have been described in prokaryotes and eukaryotes [39, 40]. The RapH phosphatase acts both on Spo0F to inhibit the sporulation phosphorelay and on ComA to inhibit its binding to DNA [41]. However, NprR is the first example of a quorum-sensor that recognizes DNA for its transcription factor activity and a protein for its sporulation inhibitor activity. This bifunctional regulator may have been selected in *B*. *cereus* group because it provides benefits to a pathogenic bacterium feeding on host proteins. The pathogenic lifestyle may require close coordination between cell density, proteolytic activities and sporulation events.

## Materials and Methods

### Bacterial strains and growth conditions

The *B*. *thuringiensis* strain 407 Cry^-^ (hereafter referred to as *Bt* 407 strain) is an acrystalliferous strain cured of its *cry* plasmid [42]. *Escherichia coli* K-12 strains TG1 was used as host for the construction of plasmids and cloning experiments. Plasmid DNA for *Bacillus* electroporation was prepared from the Dam^-^ Dcm^-^ *E*. *coli* strain ET12567 (Strata gene, La Jolla, CA, USA). *E*. *coli* and *B*. *thuringiensis* cells were transformed by electroporation as described previously [42, 43]. *E*. *coli* strains were grown at 37°C in Luria Broth (LB). *Bacillus* strains were grown at 30°C in LB or at 37°C in HCT, a sporulation-specific medium [44].

The following concentrations of antibiotic were used for bacterial selection: ampicillin 100 μg/ml for *E*. *coli*; tetracycline 10 μg/ml, spectinomycin 200 μg/ml and erythromycin 10 μg/ml for *B*. *thuringiensis*. Bacteria with the Lac^+^ phenotype were identified on LB plates containing 100 μg/ml X-gal. The *xylA* promoter in *B*. *thuringiensis* was induced by adding 20 mM xylose to the culture medium.

### Sporulation assays

The sporulation efficiency of the *B*. *thuringiensis* strains was determined in LB medium after 3 days of growth. Numbers of viable cells were counted as total colony-forming units (cfu) on LB plates. The number of spores was determined as heat-resistant (65°C for 30 min) cfu on LB plates. The percentage of sporulation was calculated as 100 × the ratio between the numbers of heat-resistant spores ml^−1^ and viable bacteria ml^−1^. The OD_600_ at the onset of the stationary phase is similar for all tested strains (ranging from 2.1 to 2.6). Results are given as mean ± standard error of the mean (SEM). The experimental values are given in S1 Table.

### DNA manipulations

Chromosomal DNA was extracted from *B*. *thuringiensis* cells using the Puregene DNA Purification Kit (QIAgen, France). Plasmid DNA was extracted from *E*. *coli* by a standard alkaline lysis procedure using QIAprep spin columns (QIAgen, France). DNA polymerase, restriction enzymes and T4 DNA ligase (New England Biolabs, USA) were used in accordance with the manufacturer’s recommendations. Oligonucleotide primers (S2 Table) were synthesized by Sigma-Proligo (France). PCRs were performed in an Applied Biosystem 2720 Thermak cycler (Applied Biosystem, USA). Amplified fragments were purified using the QIAquick PCR purification Kit (QIAgen, France). Digested DNA fragments were separated on 1% agarose gels and extracted from gels using the QIAquick gel extraction Kit (QIAgen, France). Nucleotide sequences were determined by Beckman Coulter Genomics (Takeley, U K).

### Plasmid constructions

The plasmid pRN5101 [45] was used for *nprX* disruption. The plasmid pMAD-*amy*::*spc* [38] was used for complementation experiment at the *amy* locus. Transcriptional fusions for the *spoIIE* promoter region was constructed in pHT304-18’*lacZ* [46]. The low-copy-number plasmid pHT304.18-P_*xylA*_ [15] was used for complementation experiment with the modified *spo0A*_*sad67*_ gene under xylose-inducible promoter. The vectors pQE60 and pQE30 (QIAgen, France) were used to overproduce NprR-6His and 6His-Spo0F from *Bt* 407, respectively. The pQE30 was also used to overproduce 6His-Spo0F and 6His-KinA from *Bacillus subtilis* for NprR Spo0F-P dephosphorylation assays. All the constructed plasmids used in this study are described in S3 Table.

### Construction of the *Bacillus* 407 recombinant strains

The thermosensitive plasmid pRN5101Ω*nprX*::*tet* (S3 Table) was used to disrupt the chromosomal wild-type copy of *nprX* by homologous recombination as described previously [47]. The recombinant strain, designated *Bt* 407 ΔX, was resistant to tetracycline and sensitive to erythromycin. The *Bt* 407 *nprR-nprX*::*tet* (*Bt 407* ΔRX), *Bt* 407 *nprR*::*tet* (*Bt 407* ΔR) and *Bt* 407 *spo0A*::*kan* (*Bt 407* Δ0A) mutant strains were described previously [8, 33].

### ß-Galactosidase assays

ß-Galactosidase activities were measured as described previously [8], and specific activities are expressed in units of ß-galactosidase per milligram of protein (Miller units). Each assay was independently repeated at least three times and a representative graph was shown for each experiment.

### Protein preparation

The (ΔHTH)(Y223A/F225A) mutant of the *nprR* gene from *B*. *thuringiensis* strain 407 has been cloned into a pQE60 plasmid and expressed in *E*. *coli* strain M15 [pRep4] with a C-terminal 6xHis tag. The recombinant protein was purified as already described for the full-length wild-type NprR [8]. The purified protein was aliquoted, flash frozen and stored at -20°C in 20 mM Tris-HCl pH8 and 100 mM NaCl.

Selenomethionine-labelled NprR(ΔHTH))(Y223A/F225A) was produced in *E*. *coli* strain M15 [pREP4, pQE60Ω*nprR*ΔHTH-Y223A-F225A] grown at 37°C in the presence of 50 μg/ml ampicillin and 50 μg/ml kanamycin in M63 medium. At OD_600nm_ of 1 the M63 medium was supplemented with 10 g/l of Studier amino acid and 2.5 g/l of selenomethionine. Finally, expression was induced at OD_600nm_ of 1.5 by adding 1 mM IPTG and the culture was incubated for 4 hours at 37°C. The labelled protein was purified following the same protocol as for the unlabelled protein.

Spo0F from *B*. *thuringiensis* was produced as an N-terminal His-tagged recombinant protein in *E*. *coli* strain NMA522 [pQE30Ωspo0F] grown at 37°C in the presence of 50 μg/ml of ampicillin. Expression was induced at OD_600nm_ of 0.6 by adding 1 mM IPTG and the culture was incubated for 3 hours at 37°C. The protein was produced in inclusion bodies, inclusion bodies were resuspended in 25 mM Tris-HCl pH 8, 500 mM NaCl, 3 M Guanidinium Chloride and 10% Glycerol and incubated for 3 hours at 4°C. Finally Spo0F was renatured on the IMAC column following the protocol of the manufacturer. The purification was completed by a size exclusion chromatography step using a S75 16/60 column equilibrated in 20 mM Tris-HCl pH 8, 200 mM NaCl, 10% Glycerol.

Spo0F and KinA from *B*. *subtilis* were produced as N-terminal His-tagged recombinant proteins in the *E*. *coli* M15 [pRep4] strain grown at 37°C in the presence of 50 μg/ml ampicillin and 50 μg/ml kanamycin. Expression was induced at OD_600nm_ of 0.6 by adding 1 mM IPTG and the cultures were incubated overnight at 15°C. The *Bs* Spo0F and KinA proteins were then purified using an IMAC column following the protocol of the manufacturer (QIAGEN). The purification was completed by a size exclusion chromatography step using a S75 16/60 and a S200 16/60 column, for Spo0F and KinA respectively. The samples were stored frozen in 50 mM Tris-HCl pH 7.5, 50 mM Na_2_SO_4_, 15% Glycerol, 5 mM MgCl_2_ for Spo0F and KinA.

### Interaction measurements

Microscale thermophoresis (MST) [48] was performed using the Monolith NT.115 apparatus and MST Premium coated capillaries from Nanotemper Technologies. NprR was fluorescently labelled with the blue fluorescent dye NT-495-NHS according to the manufacturer’s protocol. The *Bt* Spo0F solutions were serially diluted from about 260 μM to 8 nM in the presence of 33 nM labelled NprR and 5% glycerol in MST buffer (50 mM Tris-HCl, pH 7.4, 150 mM NaCl, 10 mM MgCl_2_, 0.05% Tween-20). Data analyses were performed using the Nanotemper Analysis software.

### *In vitro* phosphorylation assay

Phosphorylation of *B*. *subtilis* Spo0F was determined in a reaction buffer (50 mM Tris-HCl, pH 7.4, 20 mM MgCl**2**, 0.1 mM EDTA and 5% glycerol). KinA (0.1 μM) was first activated by pre-incubation (1.5 hours at 37°C) in the presence of 1 mM ATP, containing 20 μCi mmol^-1^ [γ-32P]-ATP. Spo0F (5.4 μM), NprR (10 μM) and NprX-8 (57 μM) were then added as indicated, and mixtures were further incubated at 37°C. Aliquots were withdrawn at different times (2, 10 and 20 minutes). Samples were analyzed by 15% SDS-Tris-glycine PAGE. The gel was dried and the radioactivity detected with a PhosphorImager and the ImageQuant software (Molecular Dynamics Corp.).

### Protein crystallization

In order to determine the conformation of NprR carrying out the sporulation inhibitor function of the protein, we performed a large-scale screening of crystallization conditions. The truncated double mutant protein NprR(ΔHTH)(Y223A/F225A) crystallized at 18°C in 1.0 M Na citrate, 0.1 M Hepes pH 7.6. Initial needle clusters were improved using micro- and macro seeding. The crystals were flash frozen in the crystallization solution supplemented with 30% glycerol for data collection.

### Crystal structure determination

A native data set was collected at 3.25Å resolution on beamline ID29-1 (ESRF, Grenoble, France). A selenomethionine (SeMet) labelled form of the protein was used to collect anomalous diffraction data on beamline Proxima-1 (SOLEIL, Gif-sur-Yvette, France). The derivative data set was collected at a wavelength of 0.97911 corresponding to the maximum of anomalous dispersion f”. Both data sets were processed with the XDS package [49]. Sub-structure determination was performed with the SHELX program suite [50] using the HKL2MAP interface [51]. The positions of 16 from the 22 expected selenium atoms could be identified and helped determine initial crystallographic phases using the program PHASER [52]. The PHENIX wizard [53] was then used for iterative model building, density modification and structure refinement. The final model was refined against the 3.25Å resolution native data set and manually optimized using COOT [54]. Data processing and refinement statistics are given in S4 Table.

### Structure analysis

We used the Protein structure comparison service PDBeFold at European Bioinformatics Institute [55], to superimpose and compare crystal structures. We used the Protein Interfaces, Surfaces and Assemblies service PISA at the European Bioinformatics Institute [36] to analyze interactions. We used the PyMOL Molecular Graphics System [56] to analyze the 3D structures and prepare Figs 4A, 4C, 5B, 5C, S3 and S5.

### Accession numbers

The genome of *Bacillus thuringiensis* 407 used in this study is accessible in the NCBI Reference Sequence (RefSeq) Database under number NC_018877.1.

## Supporting Information

S1 Fig Mutated MTQ motif of the HTH domain. (A) Sequence alignment of the HTH domains of NprR and PlcR. The position of the mutated MTQ motif of NprR* is underlined in blue. (B) Analysis of the PlcR/DNA interactions (PDB ID 3U3W). PlcR residues L19, T20 and Q21 from PlcR, equivalent to the MTQ motif of NprR, are highlighted in sticks and labelled.
(TIF)

S2 Fig Transcription activity assays. (A) NprR* lacking a functional HTH domain is unable to activate *nprA* transcription. β-Galactosidase activity of the *Bt* 407 wild type strain (RX PnprA’Z) and the Bt 407 ΔRX *amy*::nprR*-*nprX* mutant strain (R*X PnprA’Z) harbouring the pHT304.18-*nprA*’*Z* plasmid. (B) Effect of NprR single mutations Y223A and F225A on *nprA* transcription. β-Galactosidase activity of *Bt* 407 wild type (RX PnprA’Z) and the *Bt* 407 ΔRX *amy*::*nprR*_[Y223A]_-*nprX* (R[Y223A]-X PnprA’Z) and *Bt* 407 ΔRX *amy*::*nprR*_[F225A]_-*nprX* (R[F225A]-X PnprA’Z) mutant strains harbouring the pHT304.18-*nprA*’*Z* plasmid. (C) Effect of NprR single mutations Y165A and R343A on *nprA* transcription. β-Galactosidase activity of *Bt* 407 wild type (RX PnprA’Z) and the *Bt* 407 ΔRX *amy*::*nprR*_[Y165A]_-*nprX* (R[Y165A]-X PnprA’Z) and *Bt* 407 ΔRX *amy*::*nprR*_[R343A]_-*nprX* (R[R343A]-X PnprA’Z) mutant strains harbouring the pHT304.18-*nprA*’*Z* plasmid. The cells were grown at 37°C in HCT medium. Time zero was defined as the onset of the stationary phase.
(TIF)

S3 Fig Structure of the Rap proteins. (A) Flexibility of the apo form. Apo forms of the Rap proteins. Apo-RapI (PDB ID 4I1A) and apo RapF (PDB ID 4I9E) structures. (B) Conserved dimerization mode of the Rap proteins. Closed TPR conformation of the RapF-PhrC complex (PDB ID 4I9C) and 3-helix bundle conformation of apo RapF (PDB ID 4I9E). The protein chains are shown as cartoon with cylindrical helices and coloured by spectrum from blue (Nter) to red (Cter). The conserved C-terminal type-II interface is highlighted by dots surrounding residue F360 equivalent to the NprR residue Y410. The bound peptides are displayed as black sticks.
(TIF)

S4 Fig Structure based-sequence alignments between RapF, RapH and NprR. The TPR motifs are highlighted by arrows coloured by spectrum from blue to red. Conserved NprR residues D107, Y118, E188 and Y223 involved in Spo0F binding are highlighted by red stars. Residues Y165, W167, H201, H205, H206 and R343 are indicated by green triangles. Structural data from PDB files 4I9C (RapF [20]), 3Q15 (RapH [18]) and 4GPK (NprR [22]).
(TIF)

S5 Fig Role of conserved Rap residues. (A) Role of residues Y158, F159, R115 and Y117 in the dimerization interface of RapF 3-helix bundle conformation (PDB ID 4I9E). (B) Position of residues Y158 and F159 in the dimerization interface of RapF TPR conformation (PDB ID 4I9C). (C) Role of residues R115, Y117, Y153 and D297 in the TPR conformation of the RapF-PhrC complex (PDB ID 4I9C).
(TIF)

S1 Table Sporulation efficiency of *Bacillus* strains. The percentages of spores were calculated as 100 × the ratio between heat-resistant spores ml ^−1^ and viable cells ml^−1^. The viable cells and heat-resistant spores were counted after 3 days in LB medium at 30°C. OD_600_ at t0 corresponds to the OD_600_ at the onset of the stationary phase. n is the number of independent sporulation efficiency measurements. Nd: Not detected. Results are given as mean ± standard error of the mean (SEM).
(DOCX)

S2 Table Primers used in this study. The restriction sites are underlined. Bases in red correspond to the point mutations.
(DOCX)

S3 Table Plasmids constructed for this study. References given in S3 Table following manuscript numbering.
(DOCX)

S4 Table X-ray data processing and refinement statistics. (DOCX)

## References

[1] Hilbert DW, Piggot PJ. Compartmentalization of gene expression during Bacillus subtilis spore formation. Microbiol Mol Biol Rev. 2004;68(2):234–62. 15187183 10.1128/MMBR.68.2.234-262.2004 PMC419919

[2] Rutherford ST, Bassler BL. Bacterial quorum sensing: its role in virulence and possibilities for its control. Cold Spring Harb Perspect Med. 2012;2(11):a012427 10.1101/cshperspect.a012427 23125205 PMC3543102

[3] Slamti L, Perchat S, Huillet E, Lereclus D. Quorum sensing in Bacillus thuringiensis is required for completion of a full infectious cycle in the insect. Toxins. 2014;6(8):2239–55. 10.3390/toxins6082239 25089349 PMC4147580

[4] Declerck N, Bouillaut L, Chaix D, Rugani N, Slamti L, Hoh F, et al Structure of PlcR: Insights into virulence regulation and evolution of quorum sensing in Gram-positive bacteria. Proc Natl Acad Sci U S A. 2007;104(47):18490–5. 17998541 10.1073/pnas.0704501104 PMC2141804

[5] Perego M, Hoch JA. Cell-cell communication regulates the effects of protein aspartate phosphatases on the phosphorelay controlling development in Bacillus subtilis. Proc Natl Acad Sci U S A. 1996;93(4):1549–53. 8643670 10.1073/pnas.93.4.1549 PMC39978

[6] Solomon JM, Lazazzera BA, Grossman AD. Purification and characterization of an extracellular peptide factor that affects two different developmental pathways in Bacillus subtilis. Genes Dev. 1996;10(16):2014–24. 8769645 10.1101/gad.10.16.2014

[7] Lereclus D, Agaisse H, Gominet M, Salamitou S, Sanchis V. Identification of a Bacillus thuringiensis gene that positively regulates transcription of the phosphatidylinositol-specific phospholipase C gene at the onset of the stationary phase. J Bacteriol. 1996;178(10):2749–56. 8631661 10.1128/jb.178.10.2749-2756.1996 PMC178008

[8] Perchat S, Dubois T, Zouhir S, Gominet M, Poncet S, Lemy C, et al A cell-cell communication system regulates protease production during sporulation in bacteria of the Bacillus cereus group. Mol Microbiol. 2011;82(3):619–33. 10.1111/j.1365-2958.2011.07839.x 21958299

[9] Bae T, Kozlowicz BK, Dunny GM. Characterization of cis-acting prgQ mutants: evidence for two distinct repression mechanisms by Qa RNA and PrgX protein in pheromone-inducible enterococcal plasmid pCF10. Mol Microbiol. 2004;51(1):271–81. 14651627 10.1046/j.1365-2958.2003.03832.x

[10] Shi K, Brown CK, Gu ZY, Kozlowicz BK, Dunny GM, Ohlendorf DH, et al Structure of peptide sex pheromone receptor PrgX and PrgX/pheromone complexes and regulation of conjugation in Enterococcus faecalis. Proc Natl Acad Sci U S A. 2005;102(51):18596–601. 16339309 10.1073/pnas.0506163102 PMC1317922

[11] Parashar V, Aggarwal C, Federle MJ, Neiditch MB. Rgg protein structure-function and inhibition by cyclic peptide compounds. Proc Natl Acad Sci U S A. 2015;112(16):5177–82. 10.1073/pnas.1500357112 25847993 PMC4413276

[12] Blatch GL, Lassle M. The tetratricopeptide repeat: a structural motif mediating protein-protein interactions. Bioessays. 1999;21(11):932–9. 10517866 10.1002/(SICI)1521-1878(199911)21:11<932::AID-BIES5>3.0.CO;2-N

[13] D'Andrea LD, Regan L. TPR proteins: the versatile helix. Trends Biochem Sci. 2003;28(12):655–62. 14659697 10.1016/j.tibs.2003.10.007

[14] Perego M, Brannigan JA. Pentapeptide regulation of aspartyl-phosphate phosphatases. Peptides. 2001;22(10):1541–7. 11587783 10.1016/s0196-9781(01)00490-9

[15] Slamti L, Lereclus D. A cell-cell signaling peptide activates the PlcR virulence regulon in bacteria of the Bacillus cereus group. EMBO J. 2002;21(17):4550–9. 12198157 10.1093/emboj/cdf450 PMC126190

[16] Aravind L, Anantharaman V, Balaji S, Babu MM, Iyer LM. The many faces of the helix-turn-helix domain: transcription regulation and beyond. FEMS Microbiol Rev. 2005;29(2):231–62. 15808743 10.1016/j.femsre.2004.12.008

[17] Baker MD, Neiditch MB. Structural basis of response regulator inhibition by a bacterial anti-activator protein. PLoS Biol. 2011;9(12):e1001226 10.1371/journal.pbio.1001226 22215984 PMC3246441

[18] Parashar V, Mirouze N, Dubnau DA, Neiditch MB. Structural basis of response regulator dephosphorylation by Rap phosphatases. PLoS Biol. 2011;9(2):e1000589 10.1371/journal.pbio.1000589 21346797 PMC3035606

[19] Parashar V, Jeffrey PD, Neiditch MB. Conformational change-induced repeat domain expansion regulates rap phosphatase quorum-sensing signal receptors. PLoS Biol. 2013;11(3):e1001512 10.1371/journal.pbio.1001512 23526881 PMC3601965

[20] Gallego Del Sol F, Marina A. Structural basis of rap phosphatase inhibition by phr peptides. PLoS Biol. 2013;11(3):e1001511 10.1371/journal.pbio.1001511 23526880 PMC3601957

[21] Dubois T, Faegri K, Perchat S, Lemy C, Buisson C, Nielsen-LeRoux C, et al Necrotrophism is a quorum-sensing-regulated lifestyle in Bacillus thuringiensis. PLoS Pathog. 2012;8(4):e1002629 10.1371/journal.ppat.1002629 22511867 PMC3325205

[22] Zouhir S, Perchat S, Nicaise M, Perez J, Guimaraes B, Lereclus D, et al Peptide-binding dependent conformational changes regulate the transcriptional activity of the quorum-sensor NprR. Nucleic Acids Res. 2013;41(16):7920–33. 10.1093/nar/gkt546 23793817 PMC3763537

[23] Ishikawa S, Core L, Perego M. Biochemical characterization of aspartyl phosphate phosphatase interaction with a phosphorylated response regulator and its inhibition by a pentapeptide. J Biol Chem. 2002;277(23):20483–9. 11923303 10.1074/jbc.M201086200

[24] Bongiorni C, Stoessel R, Shoemaker D, Perego M. Rap phosphatase of virulence plasmid pXO1 inhibits Bacillus anthracis sporulation. J Bacteriol. 2006;188(2):487–98. 16385039 10.1128/JB.188.2.487-498.2006 PMC1347315

[25] Burbulys D, Trach KA, Hoch JA. Initiation of sporulation in B. subtilis is controlled by a multicomponent phosphorelay. Cell. 1991;64(3):545–52. 1846779 10.1016/0092-8674(91)90238-t

[26] Sonenshein AL. Control of sporulation initiation in Bacillus subtilis. Curr Opin Microbiol. 2000;3(6):561–6. 11121774 10.1016/s1369-5274(00)00141-7

[27] Cabrera R, Rocha J, Flores V, Vazquez-Moreno L, Guarneros G, Olmedo G, et al Regulation of sporulation initiation by NprR and its signaling peptide NprRB: molecular recognition and conformational changes. Appl Microbiol Biotechnol. 2014;98(22):9399–412. 10.1007/s00253-014-6094-8 25256619

[28] Dodd IB, Egan JB. Improved detection of helix-turn-helix DNA-binding motifs in protein sequences. Nucleic Acids Res. 1990;18(17):5019–26. 2402433 10.1093/nar/18.17.5019 PMC332109

[29] Grenha R, Slamti L, Nicaise M, Refes Y, Lereclus D, Nessler S. Structural basis for the activation mechanism of the PlcR virulence regulator by the quorum-sensing signal peptide PapR. Proc Natl Acad Sci U S A. 2013;110(3):1047–52. 10.1073/pnas.1213770110 23277548 PMC3549096

[30] Hoch JA. Regulation of the phosphorelay and the initiation of sporulation in Bacillus subtilis. Annual review of microbiology. 1993;47:441–65. 8257105 10.1146/annurev.mi.47.100193.002301

[31] York K, Kenney TJ, Satola S, Moran CP, Jr., Poth H, Youngman P. Spo0A controls the sigma A-dependent activation of Bacillus subtilis sporulation-specific transcription unit spoIIE. J Bacteriol. 1992;174(8):2648–58. 1556084 10.1128/jb.174.8.2648-2658.1992 PMC205905

[32] Ireton K, Rudner DZ, Siranosian KJ, Grossman AD. Integration of multiple developmental signals in Bacillus subtilis through the Spo0A transcription factor. Genes Dev. 1993;7(2):283–94. 8436298 10.1101/gad.7.2.283

[33] Lereclus D, Agaisse H, Gominet M, Chaufaux J. Overproduction of encapsulated insecticidal crystal proteins in a Bacillus thuringiensis spo0A mutant. Bio/technology. 1995;13(1):67–71. 9634751 10.1038/nbt0195-67

[34] Perego M. A peptide export-import control circuit modulating bacterial development regulates protein phosphatases of the phosphorelay. Proc Natl Acad Sci U S A. 1997;94(16):8612–7. 9238025 10.1073/pnas.94.16.8612 PMC23044

[35] Jiang M, Grau R, Perego M. Differential processing of propeptide inhibitors of Rap phosphatases in Bacillus subtilis. J Bacteriol. 2000;182(2):303–10. 10629174 10.1128/jb.182.2.303-310.2000 PMC94277

[36] Krissinel E, Henrick K. Inference of macromolecular assemblies from crystalline state. J Mol Biol. 2007;372(3):774–97. 17681537 10.1016/j.jmb.2007.05.022

[37] Lereclus D, Agaisse H, Grandvalet C, Salamitou S, Gominet M. Regulation of toxin and virulence gene transcription in Bacillus thuringiensis. International journal of medical microbiology: IJMM. 2000;290(4–5):295–9. 11111901 10.1016/S1438-4221(00)80024-7

[38] Verplaetse E, Slamti L, Gohar M, Lereclus D. Cell Differentiation in a Bacillus thuringiensis Population during Planktonic Growth, Biofilm Formation, and Host Infection. mBio. 2015;6(3):e00138–15. 10.1128/mBio.00138-15 25922389 PMC4436061

[39] Jeffery CJ. Multifunctional proteins: examples of gene sharing. Annals of medicine. 2003;35(1):28–35. 12693610 10.1080/07853890310004101

[40] Commichau FM, Stulke J. Trigger enzymes: bifunctional proteins active in metabolism and in controlling gene expression. Mol Microbiol. 2008;67(4):692–702. 18086213 10.1111/j.1365-2958.2007.06071.x

[41] Smits WK, Bongiorni C, Veening JW, Hamoen LW, Kuipers OP, Perego M. Temporal separation of distinct differentiation pathways by a dual specificity Rap-Phr system in Bacillus subtilis. Mol Microbiol. 2007;65(1):103–20. 17581123 10.1111/j.1365-2958.2007.05776.x

[42] Lereclus D, Arantes O, Chaufaux J, Lecadet M. Transformation and expression of a cloned delta-endotoxin gene in Bacillus thuringiensis. FEMS Microbiol Lett. 1989;51(1):211–7. 2550317 10.1016/0378-1097(89)90511-9

[43] Dower WJ, Miller JF, Ragsdale CW. High efficiency transformation of E. coli by high voltage electroporation. Nucleic Acids Res. 1988;16(13):6127–45. 3041370 10.1093/nar/16.13.6127 PMC336852

[44] Lecadet MM, Blondel MO, Ribier J. Generalized transduction in Bacillus thuringiensis var. berliner 1715 using bacteriophage CP-54Ber. J Gen Microbiol. 1980;121(1):203–12. 7252480 10.1099/00221287-121-1-203

[45] Villafane R, Bechhofer DH, Narayanan CS, Dubnau D. Replication control genes of plasmid pE194. J Bacteriol. 1987;169(10):4822–9. 2443486 10.1128/jb.169.10.4822-4829.1987 PMC213861

[46] Agaisse H, Lereclus D. Structural and functional analysis of the promoter region involved in full expression of the cryIIIA toxin gene of Bacillus thuringiensis. Mol Microbiol. 1994;13(1):97–107. 7984098 10.1111/j.1365-2958.1994.tb00405.x

[47] Lereclus D, Vallade M, Chaufaux J, Arantes O, Rambaud S. Expansion of insecticidal host range of Bacillus thuringiensis by in vivo genetic recombination. Bio/technology. 1992;10(4):418–21. 1369394 10.1038/nbt0492-418

[48] Jerabek-Willemsen M, Wienken CJ, Braun D, Baaske P, Duhr S. Molecular interaction studies using microscale thermophoresis. Assay and drug development technologies. 2011;9(4):342–53. 10.1089/adt.2011.0380 21812660 PMC3148787

[49] Kabsch W. Xds. Acta crystallographica Section D, Biological crystallography. 2010;66(Pt 2):125–32. 10.1107/S0907444909047337 20124692 PMC2815665

[50] Sheldrick GM. Experimental phasing with SHELXC/D/E: combining chain tracing with density modification. Acta crystallographica Section D, Biological crystallography. 2010;66(Pt 4):479–85. 10.1107/S0907444909038360 20383001 PMC2852312

[51] Pape T, Schneider TR. HKL2MAP: a graphical user interface for phasing with SHELX programs. J Appl Cryst 2004;37:843–4.

[52] McCoy AJ, Grosse-Kunstleve RW, Adams PD, Winn MD, Storoni LC, Read RJ. Phaser crystallographic software. J Appl Cryst. 2007;40:658–74. 19461840 10.1107/S0021889807021206 PMC2483472

[53] Adams PD, Afonine PV, Bunkoczi G, Chen VB, Davis IW, Echols N, et al PHENIX: a comprehensive Python-based system for macromolecular structure solution. Acta crystallographica Section D, Biological crystallography. 2010;66(Pt 2):213–21. 10.1107/S0907444909052925 20124702 PMC2815670

[54] Emsley P, Lohkamp B, Scott WG, Cowtan K. Features and development of Coot. Acta crystallographica Section D, Biological crystallography. 2010;66(486–501). 10.1107/S0907444910007493 20383002 PMC2852313

[55] Krissinel E, Henrick K. Secondary-structure matching (SSM), a new tool for fast protein structure alignment in three dimensions. Acta crystallographica Section D, Biological crystallography. 2004;60(Pt 12 Pt 1):2256–68. 15572779 10.1107/S0907444904026460

[56] DeLano WL. The PyMOL Molecular Graphics System. on World Wide Web. 2002;http://www.pymol.org.

