# Correction: How Quorum Sensing Connects Sporulation to Necrotrophism in *Bacillus thuringiensis*

**DOI:** 10.1371/journal.ppat.1006009

**Published:** 2016-10-31

**Authors:** Stéphane Perchat, Antoine Talagas, Sandrine Poncet, Noureddine Lazar, Inès Li de la Sierra-Gallay, Michel Gohar, Didier Lereclus, Sylvie Nessler

There are two errors in Fig 3 that the publisher and authors wish to correct. During typesetting, Fig 3 was incorrectly rendered so that the top panel labels are misaligned. The publisher apologizes for the error.

Additionally, in Fig 3B gels were placed adjacent to each other without clarification of the splicing. Solid black lines have been added to indicated the splices in the corrected Fig 3B. The figure legend has also been changed to reflect the change. The authors confirm that these changes do not alter their findings. The authors have provided raw, uncropped blots as Supporting Information.

**Fig 3:**
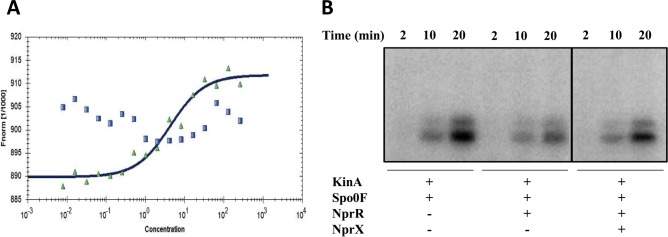
The sporulation inhibitor activity of NprR relies on Spo0F dephosphorylation. (A) Microscale thermophoresis analysis of the NprR-Spo0F interaction. The normalized NprR fluorescence F_norm_, i.e. the “hot” fluorescence divided by the “cold” fluorescence, is plotted on a linear y-axis in per mil (‰) against the total concentration of Spo0F on a log10 x-axis. The measurements were performed by using 20% LED power and 40% IR-laser power, in the presence (green triangles) and absence (blue squares) of NprX. (B) *In vitro* dephosphorylation of Spo0F-P by NprR. Purified *B*. *subtilis* Spo0F proteins (5.4 μM) was phosphorylated in a reaction containing *B*. *subtilis* KinA (0.1 μM) and [γ-32P]-ATP. Purified NprR protein and synthetic NprX-8 peptide (SSKPDIVG) were added at 10 μM and 57 μM final concentration, respectively. Time course experiments were carried out and aliquots withdrawn at the indicated time points. Black frames indicate that the figure combines two separate gels placed adjacent to each other.

## Supporting Information

S1 File Raw, uncropped blots. (ZIP)
