# Supplementary figures and images for: How Quorum Sensing Connects Sporulation to Necrotrophism in Bacillus thuringiensis
Source: PLoS Pathog. 2016 Aug 2;12(8):e1005779. doi: 10.1371/journal.ppat.1005779 (PMC4970707; doi:10.1371/journal.ppat.1005779)

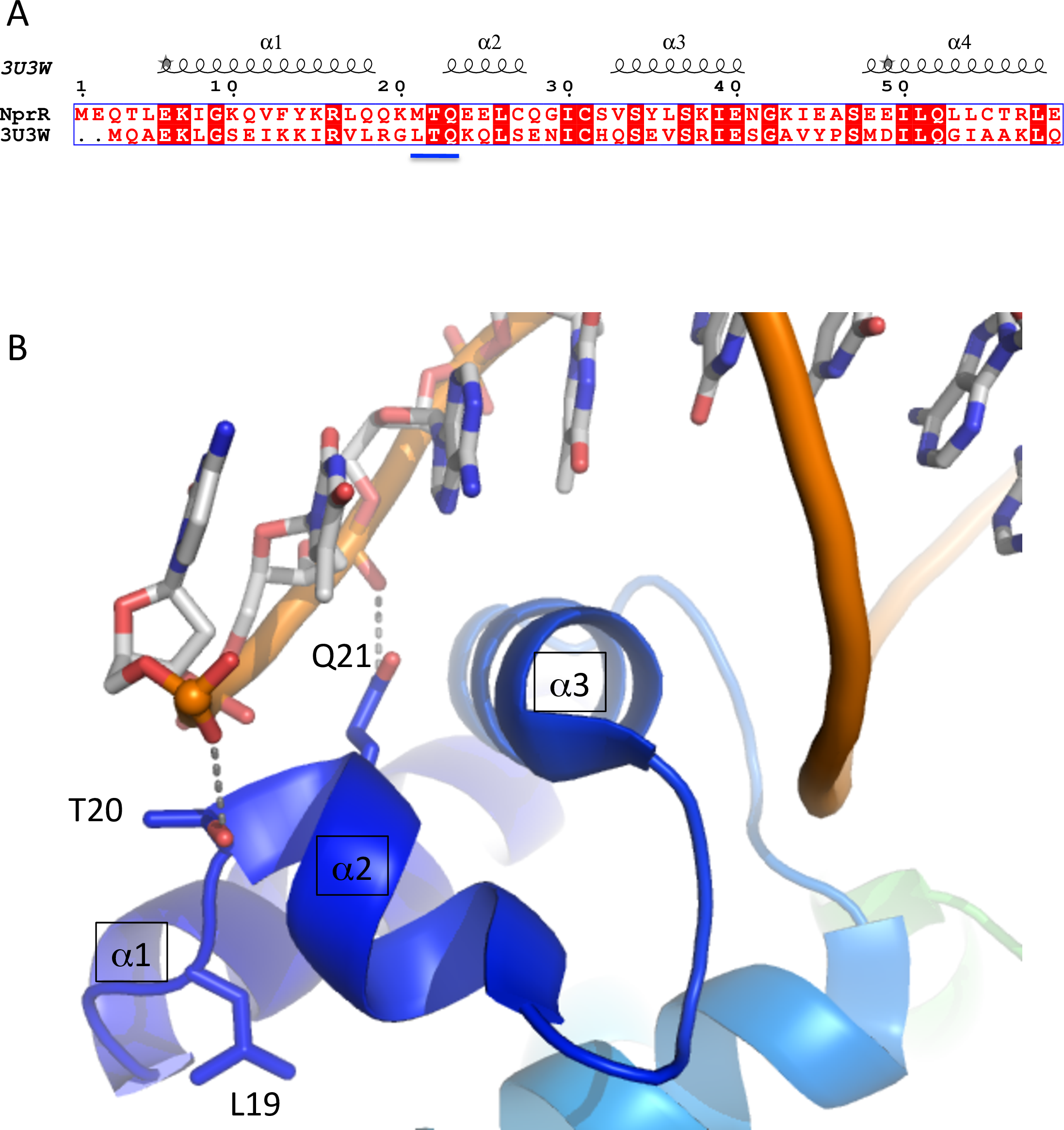

Supplement: S1 Fig — (A) Sequence alignment of the HTH domains of NprR and PlcR. The position of the mutated MTQ motif of NprR* is underlined in blue. (B) Analysis of the PlcR/DNA interactions (PDB ID 3U3W). PlcR residues L19, T20 and Q21 from PlcR, equivalent to the MTQ motif of NprR, are highlighted in sticks and labelled. (TIF) [file ppat.1005779.s001.tif]

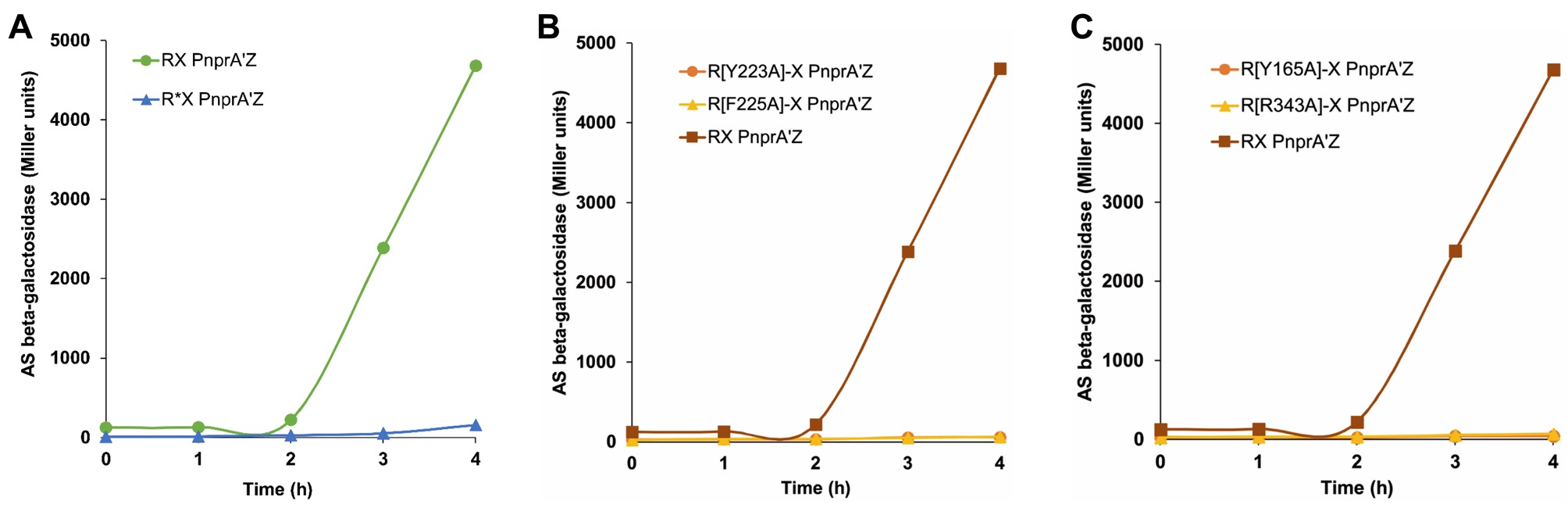

Supplement: S2 Fig — (A) NprR* lacking a functional HTH domain is unable to activate nprA transcription. β-Galactosidase activity of the Bt 407 wild type strain (RX PnprA’Z) and the Bt 407 ΔRX amy::nprR*-nprX mutant strain (R*X PnprA’Z) harbouring the pHT304.18-nprA’Z plasmid. (B) Effect of NprR single mutations Y223A and F225A on nprA transcription. β-Galactosidase activity of Bt 407 wild type (RX PnprA’Z) and the Bt 407 ΔRX amy::nprR [Y223A]-nprX (R[Y223A]-X PnprA’Z) and Bt 407 ΔRX amy::nprR [F225A]-nprX (R[F225A]-X PnprA’Z) mutant strains harbouring the pHT304.18-nprA’Z plasmid. (C) Effect of NprR single mutations Y165A and R343A on nprA transcription. β-Galactosidase activity of Bt 407 wild type (RX PnprA’Z) and the Bt 407 ΔRX amy::nprR [Y165A]-nprX (R[Y165A]-X PnprA’Z) and Bt 407 ΔRX amy::nprR [R343A]-nprX (R[R343A]-X PnprA’Z) mutant strains harbouring the pHT304.18-nprA’Z plasmid. The cells were grown at 37°C in HCT medium. Time zero was defined as the onset of the stationary phase. (TIF) [file ppat.1005779.s002.tif]

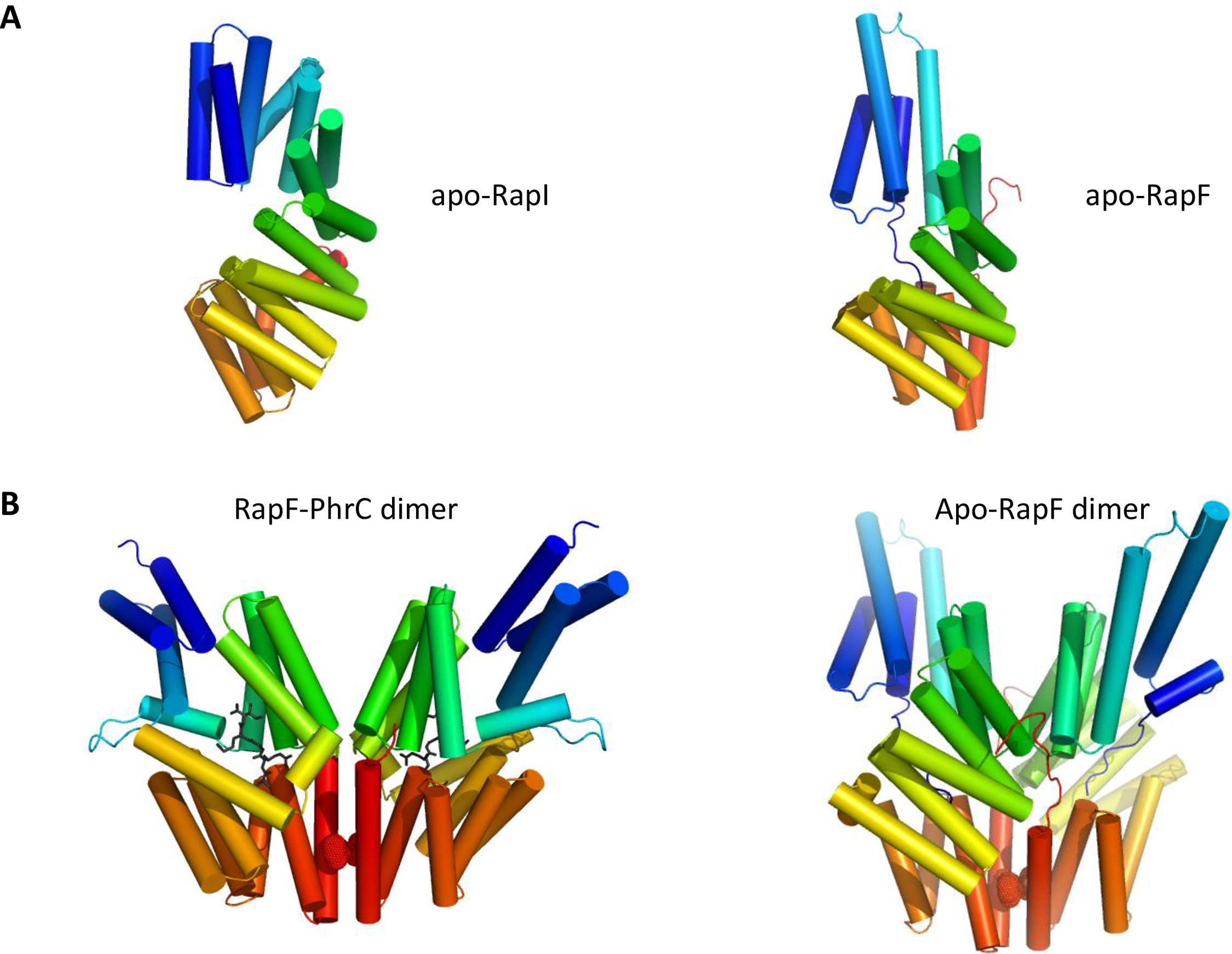

Supplement: S3 Fig — (A) Flexibility of the apo form. Apo forms of the Rap proteins. Apo-RapI (PDB ID 4I1A) and apo RapF (PDB ID 4I9E) structures. (B) Conserved dimerization mode of the Rap proteins. Closed TPR conformation of the RapF-PhrC complex (PDB ID 4I9C) and 3-helix bundle conformation of apo RapF (PDB ID 4I9E). The protein chains are shown as cartoon with cylindrical helices and coloured by spectrum from blue (Nter) to red (Cter). The conserved C-terminal type-II interface is highlighted by dots surrounding residue F360 equivalent to the NprR residue Y410. The bound peptides are displayed as black sticks. (TIF) [file ppat.1005779.s003.tif]

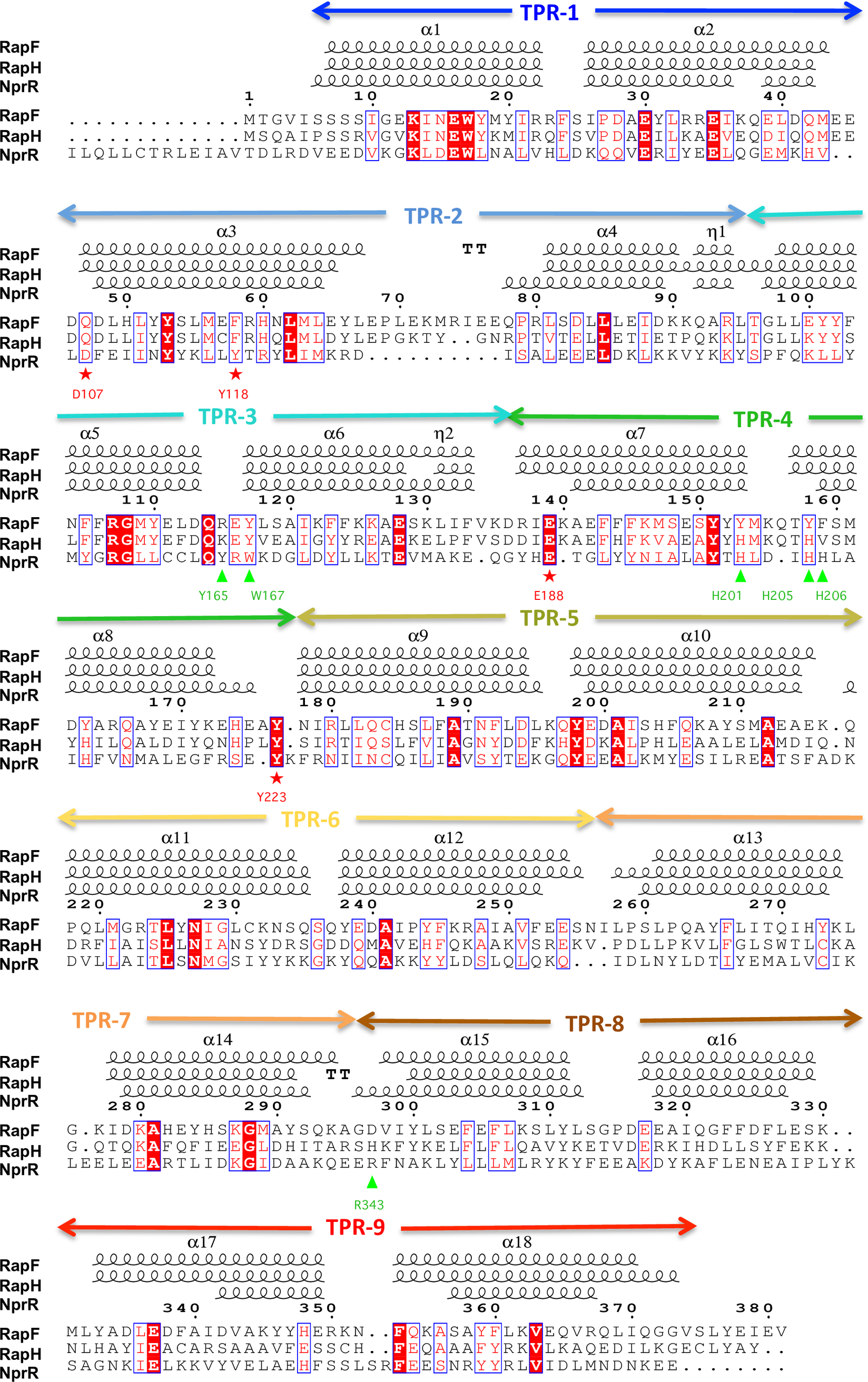

Supplement: S4 Fig — The TPR motifs are highlighted by arrows coloured by spectrum from blue to red. Conserved NprR residues D107, Y118, E188 and Y223 involved in Spo0F binding are highlighted by red stars. Residues Y165, W167, H201, H205, H206 and R343 are indicated by green triangles. Structural data from PDB files 4I9C (RapF [20]), 3Q15 (RapH [18]) and 4GPK (NprR [22]). (TIF) [file ppat.1005779.s004.tif]

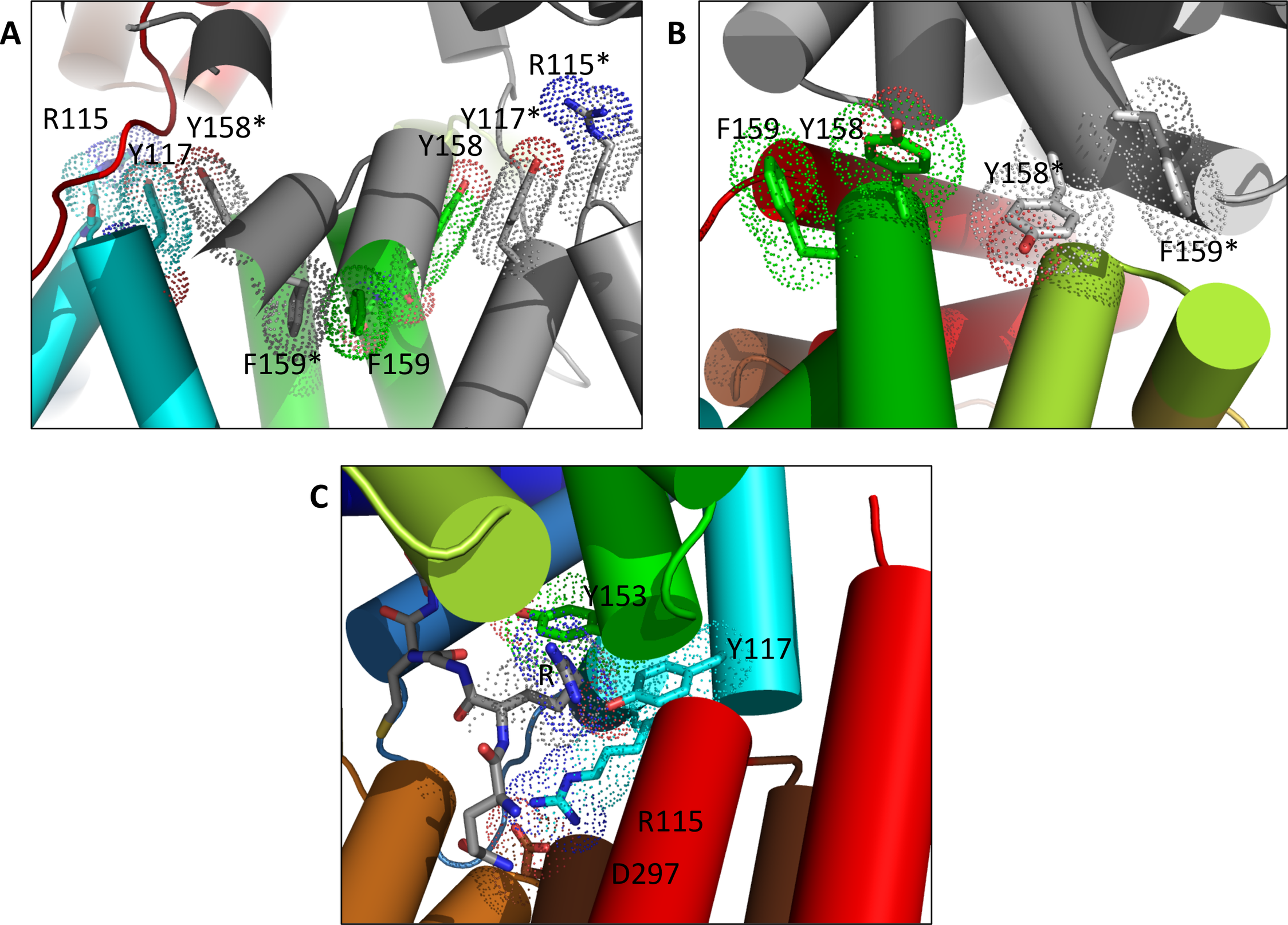

Supplement: S5 Fig — (A) Role of residues Y158, F159, R115 and Y117 in the dimerization interface of RapF 3-helix bundle conformation (PDB ID 4I9E). (B) Position of residues Y158 and F159 in the dimerization interface of RapF TPR conformation (PDB ID 4I9C). (C) Role of residues R115, Y117, Y153 and D297 in the TPR conformation of the RapF-PhrC complex (PDB ID 4I9C). (TIF) [file ppat.1005779.s005.tif]
