# Supplementary material for: How Quorum Sensing Connects Sporulation to Necrotrophism in Bacillus thuringiensis
Source: PLoS Pathog. 2016 Aug 2;12(8):e1005779. doi: 10.1371/journal.ppat.1005779 (PMC4970707; doi:10.1371/journal.ppat.1005779)
Supplement: S1 Table — The percentages of spores were calculated as 100 × the ratio between heat-resistant spores ml −1 and viable cells ml−1. The viable cells and heat-resistant spores were counted after 3 days in LB medium at 30°C. OD600 at t0 corresponds to the OD600 at the onset of the stationary phase. n is the number of independent sporulation efficiency measurements. Nd: Not detected. Results are given as mean ± standard error of the mean (SEM). (DOCX) [file ppat.1005779.s006.docx]

**Table S1:** **Sporulation efficiency of *Bacillus* strains.**

| Bacillus strains | OD_600_ at t0 | Viable counts ml^−1^ | Heat-resistant spores ml^−1^ | % spores | n |
| --- | --- | --- | --- | --- | --- |
| 407 | 2.6 ± 0.08 | 2.78E+08 ± 1.65E+07 | 1.66E+08 ± 1.16E+07 | 60 ± 1.3 | 9 |
| 407 ΔRX | 2.5 ± 0.08 | 1.68E+08 ± 2.45E+07 | 7.81E+07 ± 1.47E+07 | 46 ± 2.6 | 9 |
| 407 ΔR | 2.3 ± 0.03 | 2.00E+08 ± 1.02E+07 | 9.50E+07 ± 3.79E+06 | 48 ± 2.8 | 3 |
| 407 ΔX | 2.4 ± 0.07 | 9.27E+05 ± 3.69E+05 | 6.41E+04 ± 2.45E+04 | 7 ± 1.5 | 8 |
| 407 ΔX *amy*::X7i | 2.6 ± 0.06 | 3.03E+08 ± 1.30E+07 | 1.81E+08 ± 1.59E+07 | 60 ± 2.8 | 3 |
| 407 ΔRX *amy*::R* | 2.4 ± 0.06 | 8.76E+04 ± 5.63E+04 | 4.31E+03 ± 2.62E+03 | 5 ± 0.6 | 3 |
| 407 ΔRX *amy*::R*X | 2.5 ± 0.13 | 1.78E+08 ± 6.33E+06 | 9.40E+07 ± 1.38E+06 | 53 ± 1.1 | 3 |
| 407 Δ*spo0A* | 2.2 ± 0.12 | 2.40E+03 ± 1.47E+02 | Nd | Nd | 3 |
| 407 Δ*spo0A* [pHT-0A_67_] | 2.1 ± 0.06 | 1.60E+06 ± 2.39E+05 | 9.03E+05 ± 6.77E+04 | 56 ± 5 | 3 |
| 407 ΔX [pHT-0A_67_] | 2.3 ± 0.12 | 2.39E+08 ± 1.59E+07 | 1.37E+08 ± 5.29E+06 | 59 ± 1.9 | 3 |
| 407 ΔRX *amy*::R_[N407A/Y410A]_ | 2.6 ± 0.10 | 1.57E+08 ± 1.05E+07 | 8.35E+07 ± 3.47E+06 | 53 ± 1.4 | 3 |
| 407 ΔRX *amy*::R_[D107A]_ | 2.3 ± 0.08 | 1.74E+08 ± 5.86E+06 | 9.80E+07 ± 3.00E+06 | 56 ± 0.6 | 3 |
| 407 ΔRX *amy*::R_[Y118A]_ | 2.3 ± 0.07 | 1.91E+08 ± 3.76E+06 | 1.12E+08 ± 4.16E+06 | 59 ± 1.6 | 3 |
| 407 ΔRX *amy*::R_[E188A]_ | 2.3 ± 0.06 | 1.87E+08 ± 7.23E+06 | 1.05E+08 ± 3.61E+06 | 56 ± 1.4 | 3 |
| 407 ΔRX *amy*::R_[Y223A]_ | 2.5 ± 0.12 | 1.88E+08 ± 6.44E+06 | 1.07E+08 ± 3.21E+06 | 57 ± 2.6 | 3 |
| 407 ΔRX *amy*::R_[F225A]_ | 2.5 ± 0.12 | 1.53E+05 ± 1.49E+04 | 1.17E+04 ± 1.11E+03 | 8 ± 0.5 | 3 |
| 407 ΔRX *amy*::R_[Y165A]_ | 2.3 ± 0.08 | 1.72E+08 ± 7.09E+06 | 9.69E+07 ± 7.58E+06 | 56 ± 2.1 | 3 |
| 407 ΔRX *amy*::R_[R343A]_ | 2.2 ± 0.03 | 1.86E+08 ± 4.67E+06 | 9.87E+07 ± 6.69E+06 | 53 ± 2.3 | 3 |
| 407 ΔRX *amy*::R_[R126A]_ | 2.5 ± 0.12 | 1.84E+08 ± 7.81E+06 | 9.97E+07 ± 7.06E+06 | 54 ± 1.5 | 3 |

The percentages of spores were calculated as 100 × the ratio between heat-resistant spores ml ^−1^ and viable cells ml^−1^. The viable cells and heat-resistant spores were counted after 3 days in LB medium at 30°C. OD_600_ at t0 corresponds to the OD_600_ at the onset of the stationary phase. n is the number of independent sporulation efficiency measurements. Nd: Not detected. Results are given as mean ± standard error of the mean (SEM).
