# Supplementary material for: How Quorum Sensing Connects Sporulation to Necrotrophism in Bacillus thuringiensis
Source: PLoS Pathog. 2016 Aug 2;12(8):e1005779. doi: 10.1371/journal.ppat.1005779 (PMC4970707; doi:10.1371/journal.ppat.1005779)
Supplement: S2 Table — The restriction sites are underlined. Bases in red correspond to the point mutations. (DOCX) [file ppat.1005779.s007.docx]

**Table S2: Primers used in this study.**

| **Primer name** | **Sequence** | **Restriction site ^a^** |
| --- | --- | --- |
| Np3c | CGCGGATCCGCTGTGACGGATTTGAGAGATG | *Bam*HI |
| Np5 | TGCTCTAGAGTCAATATAAACGCCAATAC | *Xba*I |
| Np6 | CCCGCGCATGCAAGTTAATTTATAATCTCCACCC | *Sph*I |
| Np7c | CCCAAGCTTGGGTTCCTGGTAATGTAGTACGA | *Hind*III |
| Pxyl-X7i-F | CCGCTCGAGCATGTCACTATTGCTTCAGAAATACTC | *Xho*I |
| Pxyl-X7i-R | TCCCCCGGGAAACAGCTATGACCATGATTACG | *Sma*I |
| npr11X | CCGCTCGAGCATAACCGTTCTCGAATGGAAGC | *Xho*I |
| npr6fS | TCCCCCGGGTTTATTCCTCCTTATTATCATTCATT | *Sma*I |
| npr12S | TCCCCCGGGAGATTATAAATTAACTTGTTCAACAG | *Sma*I |
| mutHTH1-F | ATAAACGGCTTCAGCAAAAAGCGGCAGCAGAAGAATTATGTCAGGGCA | */* |
| mutHTH1-R | TGCCCTGACATAATTCTTCTGCTGCCGCTTTTTGCTGAAGCCGTTTAT | */* |
| PspoIIE-F | AACTGCAGCTGGCTAGAGCGTACGG | *Pst*I |
| PspoIIE-R | GCTCTAGAGCTAAAAATGCTAGCGGC | *Xba*I |
| Spo0A-Fw | cgggatccgtggagaaaattaaagtttgtg | *Bam*HI |
| Spo0A-Rv | ggggtacccatgtttaagaagccttatgctc | *Kpn*I |
| N407A/Y410A-F | CGAGATTTGAAGAAAGTGCTCGATATGCTAGGTTAGTTATTGATT | */* |
| N407A/Y410A-R | AATCAATAACTAACCTAGCATATCGAGCACTTTCTTCAAATCTCG | */* |
| D107A-F | GAAATGAAGCATGTGTTGGCTTTTGAAATTATAAATTAT | */* |
| D107A-R | ATAATTTATAATTTCAAAAGCCAACACATGCTTCATTTC | */* |
| Y118A-F | AATTATTATAAACTGCTGGCTACACGTTATTTAATTATG | */* |
| Y118A-R | CATAATTAAATAACGTGTAGCCAGCAGTTTATAATAATT | */* |
| E188A-F | AAAGAACAAGGGTATCATGCAACAGGCTTGTATTATAAT | */* |
| E188A-R | ATTATAATACAAGCCTGTTGCATGATACCCTTGTTCTTT | */* |
| Y223A-F | ATGGCATTAGAAGGATTTCGTAGTGAAGCTAAGTTTAGAAAC | */* |
| Y223A-R | GGCAATTAATTATGTTTCTAAACTTAGCTTCACTACGAAATC | */* |
| F225A-F | CATTAGAAGGATTTCGTAGTGAATATAAGGCTAGAAACATAATT | */* |
| F225A-R | CAATAAGGATTTGGCAATTAATTATGTTTCTAGCCTTATATTCAC | */* |
| Y165A-F | GATTATTGTGCTGTTTACAGGCTAGATGGAAGGATGGGCTAG | */* |
| Y165A-R | CTAGCCCATCCTTCCATCTAGCCTGTAAACAGCACAATAATC | */* |
| R343A-F | GATGCAGCAAAACAGGAAGAGGCATTTAATGCAAAATTATATTTGC | */* |
| R343A-R | GCAAATATAATTTTGCATTAAATGCCTCTTCCTGTTTTGCTGCATC | */* |
| R126A-F | CACGTTATTTAATTATGAAAGCGGATATTTCTGCACTCG | */* |
| R126A-R | CGAGTGCAGAAATATCCGCTTTCATAATTAAATAACGTG | */* |
| Npr19 | CATGCCATGGAACAAACATTAGAAAAAATAGGC | *Nco*I |
| Npr20 | GAAGATCTTTCCTCCTTATTATCATTCATT | *Bgl*II |
| spo0F-F | CGGCGGATCCATGGAAGGTAAAATTTTAATCGTTG | *Bam*HI |
| spo0F-R | GGCCGGTACCTTATGCCTCTACAGCGAGCTCATTTC | *Kpn*I |
| SAT211 | CGCGGATCCATGATGAATGAAAAAATTTTAATCG | *Bam*HI |
| SAT212 | CGGGGTACCTCAGTTAGACTTCAGG | *Kpn*I |

^a^ The restriction sites are underlined. Bases in red correspond to the point mutations.
