# Supplementary material for: How Quorum Sensing Connects Sporulation to Necrotrophism in Bacillus thuringiensis
Source: PLoS Pathog. 2016 Aug 2;12(8):e1005779. doi: 10.1371/journal.ppat.1005779 (PMC4970707; doi:10.1371/journal.ppat.1005779)
Supplement: S3 Table — References given in S3 Table following manuscript numbering. (DOCX) [file ppat.1005779.s008.docx]

**Table S3: Plasmids constructed for this study.**

| **Plasmid name** | **Plasmid features** |
| --- | --- |
| pRN5101Ω*nprX*::*tet* | 5' and 3' regions of *nprX* genes were amplified using primers Np3c/Np5 and Np6/Np7c, respectively, and 407 chromosomal DNA as template. The 5' end was purified as a *Bam*HI–*Xba*I fragment and the 3' end as a *Sph*I–*Hin*dIII fragment. The tetracycline resistance gene was purified as a *Xba*I–*Sph*I fragment from pHTS1 (Sanchis et al., 1996) and inserted with the 5' and 3' parts of *nprR-nprX* between the *Hin*dIII and *Bam*HI sites of pRN5101. |
| pHT-*nprA*’*Z* | This plasmid was previously described (Perchat et al., 2011). |
| pHT-*IIE’lacZ* | The promoter region of *spoIIE* gene was amplified using primers PspoIIE-F/PspoIIE-R with 407 chromosomal DNA as template and inserted between the *Pst*I and *Xba*I sites of pHT304.18Ω*lacZ*. This results in the creation of a transcriptional fusion between *spoIIE* promoter and *lacZ* gene. |
| pHT-*0A_67_* | The modified *spo0A_sad67_* gene was amplified using primers Spo0A-Fw/Spo0A-Rv with *B. subtilis* 168 chromosomal DNA as template and inserted between the *Bam*HI and *Kpn*I sites of the pHT304.18ΩP*_xyl_* (Slamti and Lereclus, 2002). This plasmid is a gift from L. Bouillaut. |
| pMADΩ*amy*::*spc* | This plasmid was previously described (Verplaeste et al., 2015) and used to perform chromosomal genetic complementation at the *amy* locus. The DNA fragments generated for all the constructions in the pMADΩ*amy*::*spc* were inserted between the *Xho*I and *Sma*I sites. |
| pMADΩ*amy*-*X7i* | The *X7i* gene encoding the NprX heptapeptide SKPDIVG transcribed from the xylose-inducible promoter P*_xylA_* at *amy* locus (∆X *amy*::X7i) was amplified using primers Pxyl-X7i-F/Pxyl-X7i-R from pHT1618-X7i (Perchat et al., 2011). |
| pMADΩ*amy*-*R** | Tandem M20A, T21A, Q22A substitutions in *nprR* gene were introduced using primers npr11X/npr6fS. PCR fragments amplified by primer pairs npr11X/mutHTH1-R and mutHTH1-F/npr6fS from 407 chromosomal DNA were used as template. |
| pMADΩ*amy*-*R*X* | Tandem M20A, T21A, Q22A substitutions in *nprR* gene were introduces using primers npr11X/npr12S. PCR fragments were amplified using primers npr11X/mutHTH1Rev and mutHTH1Fwd/npr12S from 407 chromosomal DNA as template. |
| pMADΩ*amy*-*R_[N407A/Y410A]_* | Tandem N407A/Y410A substitutions in *nprR* gene were introduced using primers npr11X/npr6fS. PCR fragments amplified by primer pairs npr11X/ N407A/Y410A-R and N407A/Y410A-F /npr6fS from 407 chromosomal DNA were used as template. |
| pMADΩ*amy*-*R_[D107A]_* | D107A substitution in *nprR* gene were introduced using primers npr11X/npr6fS. PCR fragments amplified by primer pairs npr11X/D107A-R and D107A-F /npr6fS from 407 chromosomal DNA were used as template. |
| pMADΩ*amy*-*R_[Y118A]_* | Y118A substitution in *nprR* gene were introduced using primers npr11X/npr6fS. PCR fragments amplified by primer pairs npr11X/Y118A-R and Y118A-F /npr6fS from 407 chromosomal DNA were used as template. |
| pMADΩ*amy*-*R_[E188A]_* | E188A substitution in *nprR* gene were introduced using primers npr11X/npr6fS. PCR fragments amplified by primer pairs npr11X/E188A-R and E188A-F /npr6fS from 407 chromosomal DNA were used as template. |
| pMADΩ*amy*-*R_[Y223A]_* | Y223A substitution in *nprR* gene were introduced using primers npr11X/npr6fS. PCR fragments amplified by primer pairs npr11X/Y223A-R and Y223A-F /npr6fS from 407 chromosomal DNA were used as template. |
| pMADΩ*amy*-*R_[Y223A]_X* | Y223A substitution in *nprR* gene were introduced using primers npr11X/npr12S. PCR fragments amplified by primer pairs npr11X/Y223A-R and Y223A-F/npr12S from 407 chromosomal DNA were used as template. |
| pMADΩ*amy*-*R_[F225A]_* | F225A substitution in *nprR* gene were introduced using primers npr11X/npr6fS. PCR fragments amplified by primer pairs npr11X/F225A-R and F225A-F  /npr6fS from 407 chromosomal DNA were used as template. |
| pMADΩ*amy*-*R_[F225A]_X* | F225A substitution in *nprR* gene were introduced using primers npr11X/npr12S. PCR fragments amplified by primer pairs npr11X/F225A-R and F225A-F /npr12S from 407 chromosomal DNA were used as template. |
| pMADΩ*amy*-*R_[Y165A]_* | Y165A substitution in *nprR* gene were introduced using primers npr11X/npr6fS. PCR fragments amplified by primer pairs npr11X/Y165A-R and Y165A-F /npr6fS from 407 chromosomal DNA were used as template. |
| pMADΩ*amy*-*R_[Y165A]_X* | Y165A substitution in *nprR* gene were introduced using primers npr11X/npr12S. PCR fragments amplified by primer pairs npr11X/Y165A-R and Y165A-F/npr12S from 407 chromosomal DNA were used as template. |
| pMADΩ*amy*-*R_[R343A]_* | R343A substitution in *nprR* gene were introduced using primers npr11X/npr6fS. PCR fragments amplified by primer pairs npr11X/R343A-R and R343A-F /npr6fS from 407 chromosomal DNA were used as template. |
| pMADΩ*amy*-*R_[R343A]_X* | R343A substitution in *nprR* gene were introduced using primers npr11X/npr12S. PCR fragments amplified by primer pairs npr11X/R343A-R and R343A-F /npr12S from 407 chromosomal DNA were used as template. |
| pMADΩ*amy*-*R_[R126A]_* | R126A substitution in *nprR* gene were introduced using primers npr11X/npr6fS. PCR fragments amplified by primer pairs npr11X/R126A-R and R126A-F /npr6fS from 407 chromosomal DNA were used as template. |
| pQE60-*nprRΔHTH_[Y223A/F225A]_* | This plasmid was previously described (Zouhir et al., 2013)*.* |
| pQE60-*nprR_[Y223A/F225A]_* | The *nprR* gene with tandem Y223A and F225A substitutions was amplified using primers Npr19/Npr20 from pMADΩ*amy*-*R_[Y223A/F225A]_X* and inserted between the *Nco*I and *Bgl*II sites of pQE60*.* |
| pQE30-*spo0F* | The *spo0F* gene was amplified using primers spo0F-F/spo0F-R with *B. subtilis* 168 chromosomal DNA as template and inserted between the *Bam*HI and *Kpn*I sites of pQE30*.* |
| pQE30-*kinA* | The *kinA* gene was amplified using primers SAT211/SAT212 with *B. subtilis* 168 chromosomal DNA as template and inserted between the *Bam*HI and *Kpn*I sites of pQE30*.* |
