# Supplementary material for: How Quorum Sensing Connects Sporulation to Necrotrophism in Bacillus thuringiensis
Source: PLoS Pathog. 2016 Aug 2;12(8):e1005779. doi: 10.1371/journal.ppat.1005779 (PMC4970707; doi:10.1371/journal.ppat.1005779)
Supplement: S4 Table — (DOCX) [file ppat.1005779.s009.docx]

**Table S4: X-ray data processing and refinement statistics**

| Data processing statistics | Native data set | SAD data set (SeMet) |
| --- | --- | --- |
| Space group | P 21 21 21 | P 21 21 21 |
| Unit-cell parameters (Å) | a=67.8 b=105.3; c=177.1 | a=68.3; b=106.4; c=178.0 |
| Unit-cell angles (°) | α=90.0; β=90.0; γ=90.0 | α=90.0; β=90.0; γ=90.0 |
| Resolution range (Å)* | 50.0-3.24 (3.44-3.24) | 50.0-3.5 (3.59-3.50) |
| No. of unique reflections | 20 499 (3144) | 31 565 (2311) |
| Completeness (%) | 98.4 (95.8) | 99.7 (97.9) |
| Redundancy | 3.46 (3.53) | 4.46 (4.52) |
| Mean I/σ(I) | 10.03 (2.18) | 7.66 (2.09) |
| R_meas_(%)^a^ | 11.5 (50.4) | 19.7 (80.7) |
| Refinement statistics | Against native data set |  |
| Resolution range | 47.93-3.24 (3.41-3.24) |  |
| No. of molecules/a.u. | 2 |  |
| R_work_(%)^b^ | 22.0 (29.75) |  |
| R_free_(%)^c^ | 25.0 (32.87) |  |
| Ramachandran |  |  |
| Favored (%) | 93.31 |  |
| Outliers (%) | 0.57 |  |
| R.M.S.D. |  |  |
| Bond lengths (Å) | 0.004 |  |
| Bond angles (°) | 0.772 |  |
| Chirality | 0.057 |  |
| Planarity | 0.002 |  |
| Dihedral | 14.266 |  |
| Average B, all atoms (Å^2^) | 78.0 |  |

* Numbers in parentheses represent values in the highest resolution shell.

**a** Rmeas = ∑hkl [N/N-1]^1/2^∑i |Ii(hkl) - <I(hkl)>| / ∑hkl∑i Ii(hkl) where N is the multiplicity of a given reflection, Ii(hkl) is the integrated intensity of a given reflection and <I(hkl)> is the mean intensity of multiple corresponding symmetry-related reflections.

**b** Rwork = ∑ ||Fobs| - |Fcalc|| / ∑ |Fobs|, where |Fobs| and |Fcalc| are the observed and calculated structure factor amplitudes respectively.

**c** Rfree is the same as Rwork but calculated with a 20% subset of all reflections that was never used in refinement.
