# Supplementary figures and images for: Correction: How Quorum Sensing Connects Sporulation to Necrotrophism in Bacillus thuringiensis
Source: PLoS Pathog. 2016 Oct 31;12(10):e1006009. doi: 10.1371/journal.ppat.1006009 (PMC5087896; doi:10.1371/journal.ppat.1006009)

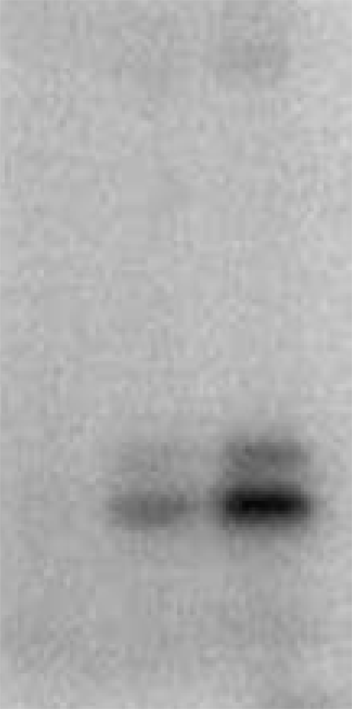

Supplement: S1 File — (ZIP) [file ppat.1006009.s001.zip › Fig3B-original right gel.tif]

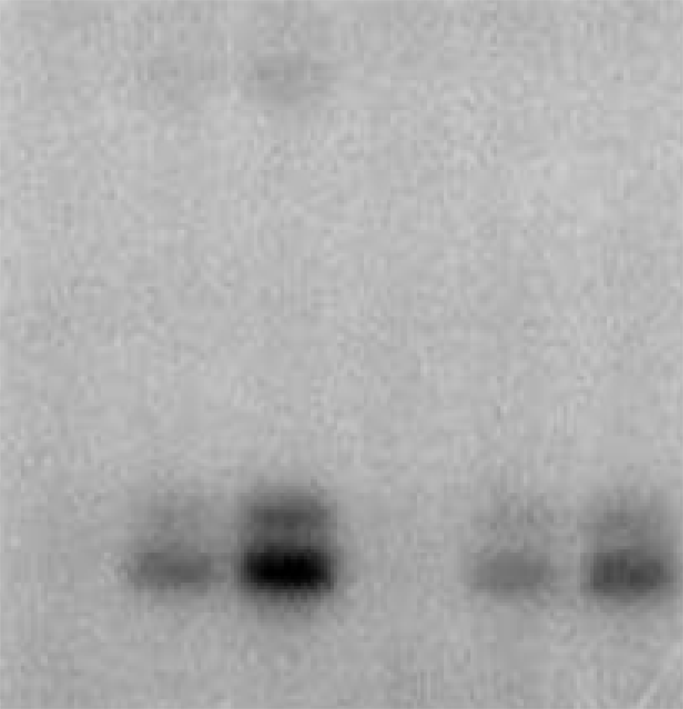

Supplement: S1 File — (ZIP) [file ppat.1006009.s001.zip › Fig3B-original left gel.tif]
